# Acculturating to multiculturalism: a new dimension of dietary acculturation among Asian American, Native Hawaiian, and Pacific Islander women in the San Francisco Bay Area, USA

**DOI:** 10.1186/s12889-024-19435-4

**Published:** 2024-08-06

**Authors:** Judy Y. Tan, Alice Guan, Autumn E. Albers, Alison J. Canchola, Laura Allen, Salma Shariff-Marco, Scarlett Lin Gomez

**Affiliations:** 1 Cancer Research Center on Health Equity, Division of Population Sciences, Cedars-Sinai Medical Center, 6500 Wilshire Blvd., Los Angeles, CA 90048 USA; 2 Department of Epidemiology and Biostatistics, University of California San Francisco, San Francisco, 94114 USA; 3 Facente Consulting, Richmond, CA 94804 USA

**Keywords:** Dietary acculturation, Asian Americans and Pacific Islanders, Multiculturalism

## Abstract

**Background:**

Dietary acculturation is the process by which diet and dietary practises from the environment of origin are retained or changed and/or those prevalent in a new environment are adopted. Despite rapid population growth the U.S., knowledge gaps exist on characterising dietary acculturation among Asian American, Native Hawaiian, and Pacific Islander communities (AANHPI). This study characterise dietary patterns in a sample representative of AANHPI on key demographic characteristics.

**Methods:**

Data were from a 2013–2014 population-based case-control study in the San Francisco Bay Area, U.S. Survey items were adapted from dietary acculturation scales developed for AANHPI populations. Validated measures assessed social capital, social standing, discrimination and immigration experiences. A principal components factor analysis was conducted to characterise dietary patterns of acculturation.

**Results:**

Three dietary patterns were identified: “Asian,” “Western,” and a distinct “Multicultural” factor. Respondents reporting a high-Asian diet tended to also report smaller social networks, higher levels of stress, and, among those born outside of the U.S., an educational standing that was better before immigration. Respondents reporting a high-Western diet tended to also report the highest level of discrimination. Those reporting a high-Multicultural diet tended to report higher neighbourhood collective efficacy.

**Conclusions:**

The finding of a distinct “Multicultural” factor beyond the typical “Asian” and “Western” factors may reflect the multidirectional relationships between culture, diet, and dietary behavior, in which origin and destination cultures interact in complex ways and where foods from multiple ethnicities intermix.

**Supplementary Information:**

The online version contains supplementary material available at 10.1186/s12889-024-19435-4.

## Background

Acculturation in the U.S. refers to the process by which a subgroup of a population, often an immigrant group, adopts characteristics of the “mainstream” U.S. culture and retains or relinquishes aspects of their “traditional” culture [1–3]. Changes in diet and dietary practises from acculturation may result from social or environmental differences that change, for instance, the availability of foods and ingredients, the cultural significance of food, or even how food is prepared and eaten [4, 5]. Dietary acculturation has been conceptualized as the process by which the diet and dietary practises prevalent in a new environment are adopted [3, 6]. Factors associated with dietary acculturation include individual characteristics (e.g., beliefs and values surrounding food, behaviours, and taste preferences), the social environment (e.g., ethnic enclaves), built environment (e.g., food availability), and socioeconomic status [3].

Despite making up approximately 7.7% of the U.S. population in 2020 [7], Asian American, Native Hawaiian, and Pacific Islander populations (AANHPI) represent an understudied group in the U.S. [8]. As dietary acculturation influences dietary behaviours, it has the potential to influence chronic disease patterns among AANHPI in the U.S. [9]. Immigration to the U.S. corresponds to a higher prevalence of chronic disease and chronic disease risk factors [10–13], with variation in this phenomenon across sociodemographic groups [14]. Compared to their counterparts residing in their country of origin, immigrants to the U.S. demonstrated a higher prevalence of type 2 diabetes [10]. People of Asian descent born in the U.S. were more likely to be overweight or obese than those born outside of the U.S. [12], with second- and third-generation AANHPI adults more likely to be obese compared to first-generation AANHPI adults [13]. Japanese American women born in the U.S. have higher percentage body fat than those who have immigrated [15]. Filipinx women in Hawaii were more likely to be obese than those living in the Philippines [11]. A “Western” diet may be associated with a greater intake of fat, anthropometric risk factors (e.g., greater waist and hip circumference, body fat and body mass index), levels of c-reactive protein (a heart disease risk factor), and type 2 diabetes [15–19].

Research has highlighted the importance of dietary acculturation, an individual-level factor, and its relationship with social and community-level factors such as discrimination and social capital, in explaining health disparities between immigrant and non-immigrant populations [20–22]. A population-based cross-sectional study with over 50,000 immigrants and individuals born in Sweden showed that, compared to Sweden-born people, being an immigrant was associated with greater psychological distress, and that social capital reduced this association [21]. In a hierarchical linear regression model that controlled for nativity of the parent, neighbourhood collective efficacy, an indicator of social capital, was a significant predictor of obesity among adolescents in Los Angeles County, U.S. [22]. Social capital and other social variables (e.g., socioeconomic status) may be confounded with dietary acculturation, since greater social power typically translates to increased purchasing power for food. However, the ways in which dietary acculturation may be influenced by social capital and other social variables remains unclear for immigrants. For example, for some immigrants from lower-income countries, while greater purchasing power in a new environment may lead to increased consumption of fruits and vegetables, it can also lead to increased consumption of “festival food” usually reserved for special occasions, such as meats, sweets, and processed food [23]. Examining the potential positive and negative associations between dietary acculturation, social capital, and other social variables is critical to characterizing how dietary acculturation may be related to health outcomes.

Research on dietary acculturation among AANHPI groups has been limited by measurement issues, including the use of two-factor measures of dietary acculturation that potentially limit our understanding of a dynamic process. Existing research on dietary acculturation among AANHPI communities has applied varied measures of dietary acculturation, and there is a lack of consensus on how to measure dietary acculturation among AANHPI people [24]. Dietary acculturation among AANHPI populations may be assessed with two scales: one that measures the maintenance of a “traditional” diet from the country or culture of origin and one that measures adoption of a “Western” diet from the country of destination [3, 17, 18]. However, people with low scores on both the “traditional” and “Western” scales are not well characterised [25]. For example, a 2018 study of dietary acculturation among ethnically Chinese people living in the U.S. found that approximately 20% of respondents had low scores on both the “Western” and “Chinese” dietary acculturation subscales, leading the authors to categorize this group as “other” [25]. There is a need to adequately describe the shift of AANHPI groups towards acculturation patterns that are neither “traditional” nor “Western” [26].

Social, environmental, and geographical contexts are critically important to understanding what determines the selective adoption and retention of cultures [26]. The San Francisco Bay Area is an urban and peri-urban landscape in which nearly two thirds of residents are people of colour and/or of mixed race and ethnicity [7]. AANHPI people make up approximately 31% of the 7.8 million Bay Area residents [7], with more than half of them born outside of the U.S. [27]. The multicultural nature of the Bay Area, along with the range of dietary options it offers as a densely populated region, makes it a prime location to understand how dietary acculturation may occur among AANHPI populations in diverse settings.

Examining dietary acculturation in specific AANHPI cultures may be illuminating given cultural differences in the significance of food and diet. Food and diet are fundamental to Chinese medicine, which uses various foods for healing and maintaining balance. Among Filipinx migrant families, preparing traditional foods at home is an important way by which parents instill Filipinx identity in children [28]. Similarly, food procurement, meal planning, and meal preparation are traditionally relegated to the role of women. As such, dietary acculturation may be a particularly salient process to examine among women. In the present study, we describe dietary patterns and their associations with social and structural characteristics for a group of AANHPI women and specifically for Chinese and Filipinx Americans residing in a region of the U.S. with a long history of AANHPI immigration, the San Francisco Bay Area, California, U.S.

## Methods

As part of the Asian American Community Health Initiative, we conducted a case-control study of breast cancer in the San Francisco-Oakland-San Jose Bay Area. The control group (i.e., women without breast cancer) from that study, which was recruited between March 2013 and October 2014, comprises the sample for the present analysis. Women were frequency-matched to cases on age- (five-year intervals) and ethnicity (Chinese, Filipinx, and other Asian American, Native Hawaiian, or Pacific Islander). The sample was recruited through targeted communications at online, community-based, and address-based sampling methods. The control sample was found to be representative of AANHPI women in the San Francisco Bay Area on key demographic characteristics, including education and marital status [29]. Thus, it is a representative sample of the source population when compared against data from the California Health Interview Survey [29, 30]. Informed consent was obtained from all of the participants. This research was approved by the Institutional Review Board of the Cancer Prevention Institute of California and the Human Research Protection Program at the University of California San Francisco (IRB study number 17-23454).

### Materials and measures

Data were collected via a one-hour telephone interview, and then participants were mailed a self-administered survey that took 20–30 min to complete (see Supplementary files for interview guide and survey). Interviews were conducted by bilingual, bicultural interviewers in Mandarin, Cantonese, Tagalog and English and all materials were available in these languages. Data collected included sample demographic characteristics such as age, nativity (U.S. or foreign-born), generational status (first, second, or third generation American if U.S.-born; age at immigration if foreign-born), English proficiency (Not good/poor, Okay/well, Very well, Only English), and income-to-poverty ratio (total annual household income divided by federal poverty thresholds based on household size).

### Dietary acculturation

To assess dietary acculturation, we used items adapted from the scales developed by Satia and colleagues [17] for Chinese American women and and validated with Filipinx [31], Vietnamese [32], Japanese [33], Korean [34–36], and Asian Indian and Pakistani [37] populations living in the U.S. For the 27 specific food and beverage items, participants were asked how often they usually consumed that item or group of items in the past 12 months with response categories recorded as never/rarely, 1–3 times per month, 1–3 times per week, 4–6 times per week, once a day, or more than once a day. Items about dietary practises included frequency of consuming homemade foods, packaged or prepared foods, eating at fast-food restaurants and restaurants serving typically non-Asian foods, eating Asian-style breakfasts and dinners, eating traditionally preserved foods, balancing yin and yang or hot and cold properties of foods (if this was part of their cultural heritage), and shopping at Asian and American supermarkets. A summary item asked respondents to characterise the “culture” of the food in their overall diet, with responses ranging from mostly Asian foods to mostly non-Asian foods. All items were included in the telephone survey except the consumption of alcohol which was included in the self-administered survey. Of the 474 women who completed the diet section, we excluded 33 participants who did not respond to questions about alcohol consumption and 1 who did not complete all items on dietary acculturation, leaving 440 (92.8%) women in the present analysis.

### Social network and status

We assessed home ownership (yes/no), number of people within social network (responses categorised into $\:\le\:$2, 3–4, or ≥5) and neighbourhood collective efficacy using five items (e.g., “How many neighbors do you know by name?”) rated on a 1–4 Likert-type scale (1, none, 4, a lot; Cronbach’s alpha=0.83) [22, 38].

### Overall stress and experiences of discrimination

To assess overall stress in the past year, we used the 10-item version of the Perceived Stress Scale rated on a Likert-type scale (1, never, 5, very often) [35]. To assess experiences with discrimination, we included eight items from the Experiences of Discrimination on how often respondents experienced discrimination across various situations in their lifetime, if at all (e.g., “How often have you been treated unfairly at work”) [36]. One item was adapted to be more contextually relevant to immigration experience (e.g., “How often have you been treated unfairly when seeking legal services related to immigration”). The number of lifetime discriminatory situations ever experienced was summarised into a single score (ranging from 0, indicating no discriminatory experiences in the lifetime to 8, indicating having experienced all eight of the discriminatory situations described). The Perceived Stress Scale (Cronbach’s alpha = 0.85) and the adapted experiences of discrimination scale (Cronbach’s alpha = 0.79) demonstrated strong reliability.

### Immigration experiences

For those born outside of the U.S., we collected data pertaining to experiences related to immigration. Respondents were asked to select the importance of any of the following reason(s) or purpose(s) for immigration: to improve life, get better education, to join family, or to find a job, which were each evaluated individually. To assess immigration-related stress, we used an adapted version of Noh’s Acculturative Stress Index, a 14-item measure of how often respondents feel that living in the U.S. is stressful due to various reasons (e.g., “because you are treated as an outsider by other Americans”) rated on a 1–4 Likert-type scale (1, never, 4, very often; Cronbach’s alpha = 0.82) [39]. We used the Subjective Social Status scale to assess perceived social standing regarding finances, education, job or occupation whether it was perceived to be better before, the same as, or better since immigrating [37]. For example, to evaluate subjective social status pretaining to money, respondents were asked, “Using the ladders below, please circle the number that corresponds to where you feel you currently stand compared to other people in the United States in terms of your money” and “Where did you stand BEFORE you came to the U.S. to live in terms of your money.” Responses ranged from 1 to 10 corresponding to the lowest and highest rungs on a ladder pictured, with higher rungs indicating being better off relative to lower rungs.

### Statistical analysis

#### Assessing factorability of the original matrix of dietary acculturation items

Bartlett’s test of sphericity and the Kaiser-Meyer-Olkin (KMO) measure of sampling adequacy (overall and individual) were used to determine if the correlation matrix of the dietary acculturation items was factorable. Specifically, the Bartlett’s test of sphericity is used to determine whether there was significant correlation between variables, and the KMO measure is used to determine the strength of the relationship between variables and patterns of correlations. Both tests provided evidence that our data displayed significant intercorrelations among variables and shared variance, indicating that it was suitable for factor analysis. The individual measures of sampling adequacy were generally very high, with 31 categories attaining measures > 0.60. This suggests that a majority of the items in the dataset exhibited strong enough correlation and shared variance with other items. Thus, we proceeded to conduct an exploratory principal components factor analysis (PCFA) to derive dietary patterns, or factors, for our sample.

PCFA, also known as exploratory factor analysis (Bryant & Yarnold, 1995), is a data reduction method used to define patterns (i.e., principal components) that included items that were correlated with each other but not with the other components. We conducted an exploratory PCFA on the correlation matrix of 39 dietary acculturation items for respondents who completed all dietary and alcohol consumption questions (*n* = 440). All food and beverage items and some dietary practises were standardised to frequency per week, while dietary practises (e.g., shopping at Asian supermarkets) reported as never/rarely, sometimes, and frequently, were coded as ordinal. The summary diet item was split into two variables, Asian food in diet and Non-Asian food in diet and coded as ordinal (i.e., the three categories of the “Asian food in diet” variable were (1) equal, more non-Asian, mostly non-Asian, (2) more Asian, and (3) mostly Asian). We selected the number of dietary factors retained in the PCFA using the following criteria: eigenvalues $\:\ge\:$ 1; scree plot construction and examination of curve break points between factors; and interpretability of the factors [38]. We used an orthogonal varimax rotation procedure to obtain simpler loading patterns. Loading weights of each of the food and beverage items and dietary practises were used to label the retained dietary factors. Each respondent had a score calculated for each dietary factor, with a higher value representing higher consumption of that dietary pattern.

Descriptive statistics were computed for all variables. We examined differences and similarities in dietary patterns by sociodemographic, general acculturation, immigration, psychosocial, and behavioural variables for the retained dietary factors. For each dietary factor, participants were grouped into tertiles for low, medium, and high consumption. We conducted cross-tabulations on each variable against tertiles of the three dietary factors and used two-tailed Chi-square tests to assess differences among tertiles by each dietary factor. Data were analysed using Stata 15 in 2019.

## Results

Table 1 shows the sample characteristics for 440 respondents. Over half were foreign-born (65.7%), married or living with a partner (66.1%), with a bachelor’s degree or higher (62.0%), 50 years or older (53.4%), and of Chinese descent (53.2%). Among those who were born outside of the U.S., 88.6% were 10 years or older at time of immigration. Among those who were U.S.-born, 47.6% were second- or third-generation.

**Table 1:** Sample characteristics (*N* = 440)

	*N*	%
**Age (years)**		
< 50	205	46.6%
50–59	131	29.8%
≥ 60	104	23.6%
**Asian Ethnicity**		
Chinese	234	53.2%
Filipinx	83	18.9%
Other Asian American^a^, Native Hawaiian or Pacific Islander	123	27.9%
**Education‡**		
< High School	75	17.0%
Some college or Associates degree	91	20.7%
Bachelor’s degree	165	37.5%
Graduate degree	108	24.5%
**Employment**		
Full-time	190	43.2%
Part-time	99	22.5%
Not working	95	21.6%
Retired	56	12.7%
**Marital Status‡**		
Married or living with partner	291	66.1%
Formerly married	67	15.2%
Single	81	18.4%
**Nativity**		
Foreign born	289	65.7%
US born	151	34.3%
**Generational status‡**		
≥ 10 years old when immigrated to US	256	59.0%
< 10 years old when immigrated to US	33	7.6%
First generation, US born	76	17.5%
Second generation, US born	29	6.7%
Third generation, US born	40	9.2%
**English proficiency**		
Not good/Poor	70	15.9%
Okay/Well	119	27.0%
Very well	59	13.4%
Only speaks English	192	43.6%

*Note*^a^ Other Asian American groups included Japanese, Korean, South Asian, Southeast Asian, “Other Asian”, and Asian mixed ethnicity. **‡** Frequencies may not add up to 440 due to refusals

PCFA identified three major dietary patterns (Table 2). Based on item loadings, Factor 1 and Factor 2 were designated “Asian” and “Western,” respectively. Factor 3 emerged as a robust factor with eight unique loadings and one item loading onto both Western and Factor 3. Factor 3 items included fruit, vegetables, tofu, dried apricots or dates, homemade food, edamame, cereal, soy nuts, and pizza or western style pasta, prompting a designation of Factor 3 as the “Multicultural” factor.

**Table 2:** Loading weights from each food, beverage, or dietary practise item per extracted factor

	“Asian”	“Western”	“Multicultural”	
Variable**	Factor 1	Factor 2	Factor 3	Uniqueness
American style supermarkets	-0.69			0.50
Non-Asian food in diet	-0.66			0.56
Packaged or prepared food such as frozen dinners or take out	-0.38	0.37		0.72
Alcohol consumption	-0.37			0.85
Non-fast-food restaurants that serve typically non-Asian food (e.g., American, Mexican, Italian restaurants)	-0.36	0.43		0.64
Think about the “hot” and “cold” characteristics of foods	0.36			0.78
Fish or fish stew	0.38			0.81
Asian-style bread, such as pan de sal and naan	0.42			0.82
Shop at [participant ethnicity] or other Asian food markets	0.62			0.56
[Participant ethnicity] or other Asian-style breakfast	0.63			0.59
Rice or rice dishes	0.68			0.52
Asian food in diet	0.69			0.49
[Participant ethnicity] or other Asian-style dinner	0.70			0.52
Butter or margarine		0.36		0.87
Cheese		0.40		0.78
Fruit Juices		0.42		0.81
Doughnuts		0.43		0.81
Pizza or American-style pasta, including spaghetti or lasagna		0.44	0.32	0.69
Chocolate or other candy		0.45		0.76
Coke or other soda		0.49		0.72
Cake, pie, or cookies		0.53		0.65
Ground beef or hamburgers		0.57		0.66
Fast-food restaurants that typically serve non-Asian food, such as McDonald’s, Subway, or Domino’s Pizza		0.58		0.61
Salty snacks		0.59		0.58
Fruit			0.59	0.64
Vegetables (not counting potatoes or light green lettuce)			0.54	0.67
Tofu			0.47	0.73
Dried apricots or dates			0.45	0.79
Homemade food or food that was prepared in your home			0.40	0.74
Edamame or soybeans			0.38	0.84
Cereal			0.38	0.79
Soy nuts			0.32	0.83
Western-style bread, rolls, or bagels				0.90
Protein or “power” bars made with soy				0.90
Black licorice				1.00
Soy milk				0.91
Whole milk, 2%, 1%, or skim milk				0.91
Tea				0.93
Foods that are fermented, pickled, or traditionally preserved				0.96

Factor loadings are only displayed for values ≥|0.3|. The “Asian” factor explained 11.3% of the variance, the “Western” factor explained 8.7% of the variance, and the “Multicultural” factor explained 6.1%, so that the total variance explained is 26.1%

* Extraction method: principal components factor analysis using varimax rotation

** Dietary factors were assessed for the past 12 months from the time of the survey

Table 3 shows the distribution of dietary patterns and consumption level by demographic characteristics. The distribution of Asian diet by consumption varied across all demographic characteristics explored. Compared to respondents who consumed a low-Asian diet, those who ate a high-Asian diet tended to be 60 years or older; ethnically Chinese or Filipinx; less educated; less likely to be employed full time; least wealthy; married/living with a partner or formerly married; foreign born and immigrated after the age of 10; and less proficient in English.

**Table 3:** Distribution of dietary acculturation factor by sociodemographic characteristics and acculturation among Asian American, native hawaiian, and Pacific Islander women (*N* = 440)*

			Asian diet				Western diet					Multicultural diet			
			Low	Medium	High		Low	Medium		High		Low	Medium	High	
Characteristic		*n*	Col %	Col %	Col %	*p*	Col %	Col %	Col %		*p*	Col %	Col %	Col %	*p*
**Age years**															
< 50		205	55.8	46.3	37.7	< 0.01	36.7	45.6	57.5		< 0.01	50.3	46.9	42.5	< 0.01
50–59		131	26.5	34.0	28.8		34.7	26.5	28.1			33.3	32.0	24.0	
60+		104	17.7	19.7	33.6		28.6	27.9	14.4			16.3	21.1	33.6	
**Ethnicity**															
Chinese		234	39.5	55.1	65.1	< 0.001	65.3	57.8	36.3		< 0.001	39.5	53.7	66.4	< 0.001
Filipinx		83	12.2	21.1	23.3		15.0	16.3	25.3			28.6	15.7	12.3	
NHPI		8	4.1	0.7	0.7		0.0	1.4	4.1			2.0	1.4	2.1	
Other Asian American^a^		115	48.2	23.1	11.0		19.7	24.5	34.3			29.9	29.3	19.2	
**Education**															
< High School		75	3.4	10.2	37.9	< 0.001	28.6	13.7	8.9		< 0.001	16.3	17.0	17.9	0.74
Some college or Associates		91	15.7	20.4	26.2		17.0	26.7	18.5			21.8	22.5	17.9	
Bachelor’s degree		165	44.2	40.8	27.6		29.3	32.9	50.7			40.8	36.7	35.2	
Graduate degree		108	36.7	28.6	8.3		25.2	26.7	21.9			21.1	23.8	29.0	
**Employment**															
Full-time		190	45.6	50.3	33.6	< 0.01	42.2	40.8	46.6		0.31	45.6	44.2	39.7	0.45
Part-time		99	27.2	17.0	23.3		21.1	22.4	24.0			25.2	19.0	23.3	
Not working		95	17.7	17.0	30.1		20.4	21.8	22.6			19.0	25.2	20.5	
Retired		56	9.5	15.6	13.0		16.3	15.0	6.8			10.2	11.6	16.4	
**Per capita household income to poverty ratio** ^**b**^															
< 1.00		131	17.7	21.1	50.7	< 0.001	33.3	29.3	26.7		0.69	29.9	27.9	31.5	0.75
1.00-1.99		63	20.4	10.9	11.6		12.9	15.6	14.4			11.6	17.0	14.4	
2.00+		176	50.3	52.4	17.1		38.8	37.4	43.8			41.5	38.8	39.7	
**Marital Status**															
Married/living w partner		291	61.9	64.6	72.4	< 0.001	62.6	72.6	63.7		0.20	61.6	63.3	74.0	0.05
Formerly married		67	9.5	18.4	17.9		17.0	15.1	13.7			13.7	17.7	14.4	
Single		81	28.6	17.0	9.7		20.4	12.3	22.6			24.7	19.1	11.6	
**Nativity**															
Foreign born		289	27.9	74.8	94.5	< 0.001	76.9	68.7	51.4		< 0.001	60.5	66.0	70.5	0.20
US born		151	72.1	25.2	5.5		23.1	31.3	48.6			39.5	34.0	29.5	
**Generational Status**															
Foreign born, age to US 10+		256	15.6	66.7	92.5	< 0.001	72.1	60.5	41.8		< 0.001	55.1	54.4	65.1	0.09
Foreign born, age to US < 10		33	12.2	8.2	2.1		4.8	8.2	9.6			5.4	11.6	5.5	
US born, 1st gen		76	35.4	14.3	2.1		10.2	16.3	25.3			20.4	17.0	14.4	
US born, 2nd gen		29	15.0	3.4	1.4		4.8	5.4	9.6			6.1	9.5	4.1	
US born, 3rd gen		40	18.4	6.8	2.1		6.8	8.2	12.3			10.9	6.1	10.3	
**English proficiency**															
Only speaks English		192	76.9	43.5	10.3	< 0.001	36.1	39.5	55.5		< 0.001	45.6	49.7	35.6	0.03
Not good/Poor		70	1.4	6.1	40.4		26.5	17.0	4.1			12.2	12.2	23.3	
Okay/Well		119	4.1	34.7	42.5		27.9	31.3	21.9			26.5	28.6	26.0	
Very well		59	17.7	15.6	6.8		9.5	12.2	18.5			15.6	9.5	15.1	

*Tertiles of dietary acculturation factors were calculated using rotated estimates; percentages within each factor are by column, not row

^a^ Other Asian American groups included Japanese, Korean, South Asian, Southeast Asian, “Other Asian”, and Asian mixed ethnicity

^b^ Income/poverty ratio < 1.00 indicates that family income was less than the poverty threshold; Income/poverty ratio between 1.00-1.99 indicates that family income was equal to or higher than poverty threshold; Income/poverty ratio 2.00 + generally indicates that the family is relatively well off

The distribution of Western diet by consumption varied across age, ethnicity, education, nativity, immigration/generational status, and English proficiency. Compared to respondents who consumed a low-Western diet, respondents who ate a high-Western diet tended to be younger than 50 years of age; ethnically Filipinx or other Asian American; college graduates; U.S.-born; and English-only speakers.

The distribution of the Multicultural diet by consumption varied across age, ethnicity, English proficiency, and only marginally across marital status and immigration/generational status. Compared to respondents who consumed a low-Multicultural diet, respondents who ate a high-Multicultural diet tended to be older than 60 years of age; ethnically Chinese; married/living with partner; foreign born and immigrated after the age of 10; and poor English speakers.

Table 4 shows the distribution of dietary patterns and consumption level by home ownership, social network, neighbourhood collective efficacy, experiences with discrimination, perceived stress; and among foreign-born respondents only, reasons for immigrating; immigration-related stress; and social standing vis-à-vis money, education, job before and after immigrating. The distribution of Asian diet by consumption varied across homeownership, size of social network, perceived stress; and among respondents born outside of the U.S., reason of immigrating and perceived social standing regarding education. Compared to respondents who consumed a low-Asian diet, those who ate a high-Asian diet tended to report not owning a home, smaller social networks (i.e., 0–2 people), higher levels of stress; and among respondents born outside of the U.S., joining family as a reason for immigrating and an educational standing that was better before immigrating. Respondents born outside of the U.S. who ate a high-Asian diet also tended to report higher levels of immigration-related stress compared to respondents born outside of the U.S. who ate a low-Asian diet.

**Table 4:** Percentage distribution of each dietary acculturation factor by social characteristics, discrimination among Asian American, native hawaiian, and Pacific Islander women (*n* = 440)*

		Asian diet				Western diet				Multicultural diet			
		Low	Medium	High		Low	Medium	High		Low	Medium	High	
Characteristic	*n*	*n* = 147	*n* = 147	*n* = 146	*p*	*n* = 147	*n* = 147	*n* = 146	*p*	*n* = 147	*n* = 147	*n* = 146	*p*
**Home ownership**													
Yes	272	68.0	70.8	47.2	< 0.001	60.3	61.0	65.1	0.66	59.2	63.7	63.5	0.67
No	166	32.0	29.3	52.8		39.7	39.0	34.9		40.8	36.3	36.6	
**Social network (# people)**													
0–2	109	14.3	26.5	33.6	< 0.001	28.6	25.9	19.9	0.41	24.5	23.8	26.0	0.61
3–4	210	50.3	44.2	48.6		44.2	45.6	53.4		50.3	50.3	42.5	
≥ 5	121	35.4	29.3	17.8		27.2	28.6	26.7		25.2	25.9	31.5	
**Neighbourhood collective efficacy**													
Lower	131	34.7	42.2	40.4	0.41	37.4	36.1	43.8	0.31	47.6	39.5	30.1	< 0.001
Moderate	137	32.0	33.3	28.1		32.7	36.1	24.7		36.7	26.5	30.1	
Higher	172	33.3	24.5	31.5		29.9	27.9	31.5		15.6	34.0	39.7	
**Experiences with discrimination** ^**1**^													
None	102	21.1	19.7	24.0	0.63	19.0	21.8	24.0	0.04	20.4	21.1	23.3	0.95
1–2	91	18.4	20.4	19.9		21.1	22.4	15.1		21.1	17.0	20.5	
3–4	152	30.6	31.3	35.6		32.0	38.1	27.4		32.0	35.4	30.1	
≥ 5	129	29.9	28.6	20.5		27.9	17.7	33.6		26.5	26.5	26.0	
**Perceived Stress Scale**													
Lower	127	41.5	24.5	19.9	< 0.001	27.9	34.0	24.0	0.24	25.9	30.6	29.5	0.11
Moderate	143	26.5	36.7	34.2		32.0	27.2	38.4		36.1	24.5	37.0	
Higher	175	32.0	38.8	45.9		40.1	38.8	37.7		38.1	44.9	33.6	
**Reason for immigration** ^**2**^ (among foreign-born)**													
Improve life	188	70.7	66.36	62.32	0.57	63.7	61.4	72.0	0.32	69.5	71.1	56.3	0.06
Better education	138	56.1	45.5	47.1	0.50	41.6	55.5	46.7	0.13	55.1	49.5	39.8	0.10
Join family	130	29.3	40.9	52.9	< 0.05	40.7	46.5	49.3	0.47	44.9	43.3	46.6	0.90
Find a job	124	36.6	48.2	40.6	0.33	39.8	42.6	48.0	0.54	43.8	44.3	40.8	0.86
Immigration-related stress (among foreign-born)**													
Lower	87	43.9	29.1	26.9	0.08	29.2	34.7	25.3	0.34	23.6	29.9	35.9	0.07
Moderate	84	34.2	30.0	26.8		24.8	28.7	26.0		23.6	32.0	31.1	
Higher	118	22.0	40.9	46.4		46.0	36.6	38.7		52.8	38.1	33.0	
Social standing regarding money (among foreign-born)**													
Better before	85	37.5	32.5	45.8	0.29	37.1	42.9	42.2	0.78	46.0	35.3	40.0	0.47
Same	66	37.5	31.2	30.5		31.5	28.6	35.6		25.4	30.9	36.3	
Better in the US	60	25.0	36.4	23.7		31.5	28.6	22.2		28.6	33.8	23.8	
Social standing regarding education (among foreign-born)**													
Better before	75	17.7	27.3	42.2	0.05	36.0	39.2	25.5	0.24	32.8	30.9	40.0	0.28
Same	81	41.2	37.7	37.2		37.1	30.4	51.1		46.3	35.3	32.5	
Better in the US	59	41.2	35.1	20.7		27.0	30.4	23.4		20.9	33.8	27.5	
Social standing regarding job/occupation (among foreign-born)**													
Better before	91	50.0	42.9	52.5	0.26	55.8	43.1	44.4	0.15	44.6	50.9	50.8	0.95
Same	49	25.0	22.9	28.7		19.5	26.2	37.8		27.7	24.6	26.2	
Better in the US	47	25.0	34.3	18.8		24.7	30.8	17.8		27.7	24.6	23.1	

*Note* Percentages represent column percentages. *Tertiles of dietary acculturation factors were calculated using rotated estimates. **Only for those who reported being foreign-born

^1^ Each of the 8 items in the Experiences with Discrimination scale was coded as “ever experienced” (i.e., 0 = never experienced; 1 = rarely, sometimes, or often experienced) and summed to reflect the total number of experiences in lifetime. Thus, summed scores ranged from 0 (none of the situations experienced) to 8 (experienced all the discriminatory situations). To facilitate interpretation, the score was categorized into 0, 1–2, 3–4, or 5 or more discriminatory situations experienced

By contrast, the distribution of Western diet by consumption varied only across experiences of discrimination. Compared to respondents who ate low-Western diet, those who ate a high-Western diet reported more lifetime experiences with discriminatory situations.

The distribution of Multicultural diet by consumption varied only across neighbourhood collective efficacy, and, among those born outside of the U.S., marginally by immigration-related stress. Compared to respondents who consumed a low-Multicultural diet, respondents who ate a high-Multicultural diet reported higher neighbourhood collective efficacy; and among those born outside of the U.S., marginally lower levels of immigration-related stress.

## Discussion

This is a cross-sectional study of dietary acculturation with 440 AANHPI women from the San Francisco Bay Area that builds upon the limited literature of dietary acculturation among AANHPI populations. A PCFA yielded three factors of dietary patterns: "Asian," "Western," and “Multicultural.” The multicultural dietary pattern is not captured by existing dietary acculturation measures which are often two dimensional (“Asian” and “Western”). The new “Multicultural” dietary factor observed in this study appears to draw upon multiple cultural influences, including dried fruit and nuts (common in Mediterranean diets), edamame (common in East and Southeast Asia), and homemade food (a “traditional” component of all cultures).

The finding of a third factor in addition to the typical “Western” and “Asian” factors in previous measures of dietary acculturation may reflect the multidirectional nature of cultural influences on diet among AANHPI women in San Francisco, U.S., in which pre- and post-immigration cultures interact in complex and dynamic ways over time in a region of the world with a relatively long Asian American history [1, 3, 9]. Among AANHPI individuals, our a three-factor dietary acculturation scale may more accurately capture the process by which diet and dietary practises from environments pre- and post-immigration influence each other over time. The “Multicultural” factor included foods from various cultures such as cereals, dried apricots and dates more typically found in western and Mediterranean diets, consistent with research that found dietary variety as a component of dietary acculturation [28]. Moreover, the “Multicultural” factor excluded fish and soy milk, which differs from a “prudent” dietary pattern often observed among Asian immigrants [40] and Asians in their countries of origin [41]. This finding of a multicultural dietary pattern distinct from an Asian or a Western pattern provides evidence of a unique immigrant experience of “bicultural” eating, in which individuals maintain traditional dietary patterns while incorporating new ones from the “host culture” [23, 42, 43]. Given the diverse environment of the nine-county San Francisco Bay Area—in which approximately 31% of residents are immigrants [27] and nearly two thirds of residents are people of colour and/or people of mixed race/ethnicity [7]—it is plausible that new dietary patterns that comprise multicultural influences emerge among AANHPI women.

The distribution patterns of “Asian” and “Western” factors by sociodemographic characteristics were consistent with findings from prior studies of acculturation among Asian immigrants. For example, respondents in the top tertile of “Western” factor were more likely to be younger than 50 years of age, college educated, and monolingual English speaking, whereas respondents in the top tertile of the “Asian” factor were more likely to be older, less educated, married, foreign-born, and less proficient in English. These findings are consistent with prior research in which Asian immigrants more adapted to a Western lifestyle were more likely to score highly on “Western” dietary acculturation scales, and those less adapted to a Western lifestyle were more likely to score highly on “Asian” dietary acculturation scales [16–18]. The distribution patterns of ”Asian” and “Western” factors by social and community-level indicators such as discrimination and social capital were also consistent with prior research. Among respondents in the top tertile of “Asian” factor, fewer respondents owned a home or had more than 5 people in their social network, and more respondents perceived greater stress and considered that their social standing regarding education was better before immigrating to the United States. Among respondents in the top tertile of “Western” factor, more respondents experienced discrimination often. Asian immigrants who retained more “traditional” or pre-immigration diet and dietary practises tended to be less wealthy, more disenfranchised, and more stressed.

Respondents in the top tertile of the “Multicultural” factor tended to be younger, married, monolingual English speaking, and to report higher neighbourhood collective efficacy compared to those in the bottom tertile. Collective efficacy measures the level of social cohesion and trust in a community and reflects a collective willingness to interact with and look out for one another [22, 38]. Larger social networks, greater neighbourhood walkability, lower stress, greater community-level support and trust, and community-level organizing are some aspects of high collective efficacy neighbourhoods [22]. While existing literature on neighbourhood collective efficacy and dietary acculturation is lacking, prior research has examined links between the neighbourhood social environment and diet-related outcomes like obesity [44], with neighbourhood collective efficacy found to be positively associated with the intake of healthy foods in some studies of other large, racially and ethically diverse urban areas of the U.S. [40, 45]. Future research is needed to examine potential mechanisms by which “traditional,” “Western,” and diets and dietary practices that incorporate a diversity of cultures may interact with social and community-level factors such as social capital and discrimination to lead to diet-related chronic diseases among AANHPI and other immigrant communities.

### Limitations

There are no comparable data on dietary practises in the U.S. or Asian countries against which to evaluate actual change in groups (or sequential data for assessing change within individuals). While some efforts were made to disaggregate AANPHI subgroups when looking at the distribution of dietary patterns across respondents, most of the data remained aggregated, potentially blurring specific subgroup associations. Aggregated data on AANHPI populations potentially blur specific subgroup associations and there is a need to disaggregate further with larger datasets. Data in this study were collected in 2013–2014; given the rapid demographic change in the San Francisco Bay Area, as well as impactful societal events (including the COVID-19 pandemic and parallel rise in attention to hate crimes against AANPHI people) that have taken place since, these data may not fully reflect the current context in which many AANPHI people live. PCFA is a relatively agnostic approach looking for similarities within the data. Although PCFA and latent class analyses are data-based and may well be population-specific, it is nevertheless a strength in the present study that the data were collected in the San Francisco Bay Area, a place that affords us looking at multiculturalism in a diverse setting in terms of AANHPI racial/ethnic composition and immigration history. Finally, we report the distribution of participants with respect to tertiles of each diet profiles; however, we did not analyse the distribution of respondents by their overall diet profile, which may have included being in the top tertile of multiple diets (e.g., having both a high-Multicultural and a high-Asian diet). Further research could explore how the demographic and social variables studied vary across diet profiles.

The study findings call for further exploration of dimensions of dietary acculturation beyond the traditional “Asian” and “Western” dimensions to capture the diversity in dietary acculturation in AANPHI and other immigrant communities. Adapting dietary acculturation measures to the rapidly evolving, multicultural environment of the U.S. will be critical to designing effective food-related policies and interventions for immigrant communities. Accuracy in assessing dietary acculturation will help elucidate the multifaceted relationships between social and structural factors and health outcomes related to diet among the growing immigrant populations in the U.S.

### Electronic supplementary material

Below is the link to the electronic supplementary material.

Supplementary Material 1

Supplementary Material 2

Supplementary Material 3

Supplementary Material 4

## Data Availability

The data underlying this article will be shared on reasonable request to the corresponding author.

## Abbreviations

AANHPI: Asian American, Native Hawaiian, Pacific Islander,
PCFA: Principal Components Factor Analysis
