# Supplementary material for: Acculturating to multiculturalism: a new dimension of dietary acculturation among Asian American, Native Hawaiian, and Pacific Islander women in the San Francisco Bay Area, USA
Source: BMC Public Health. 2024 Aug 6;24:2128. doi: 10.1186/s12889-024-19435-4 (PMC11302078; doi:10.1186/s12889-024-19435-4)
Supplement: Supplementary file 1 — Supplementary Material 1 [file 12889_2024_19435_MOESM1_ESM.docx]

**Appendix A. Factorability of the correlation matrix of the original nutrients: Bartlett's test of sphericity and Kaiser-Meyer-Olkin measure of sampling adequacy**

|  | |
| --- | --- |
| **Bartlett's test of sphericity: p-value <0.001** | |
| **Kaiser-Meyer-Olkin statistic (overall measure of sampling adequacy*): 0.78** | |
| **Individual measures of sampling adequacy*:** | |
| **<0.60** | Soy milk; Black licorice; Western-style bread, rolls, or bagels; Foods that are fermented, pickled, or traditionally preserved in other ways (such as fish sauce, kimchi, Japanese pickled vegetables, salted or preserved eggs, preserved tofu, salted fish, or Chinese sausage (lap chong)); Tea; Whole milk, 2%, 1%, or skim milk; Soy nuts |
| **0.60 to <0.80** | Edamame or soybeans; Fruit; Tofu; Doughnuts; Vegetables (not counting potatoes or light green lettuce); Butter or margarine; Cereal; Dried apricots or dates; Ground beef or hamburgers; Fruit Juices; Cake, pie, or cookies; Protein or "power" bars; Asian style bread, such as pan de sal and naan; Homemade food or food that was prepared in your home; Fast-food restaurants that typically serve non-Asian food, such as McDonald's, Subway, or Domino's Pizza; Fish or fish stew; Chocolate or other candy; Cheese; Pizza or American-style pasta, including spaghetti or lasagna; Alcohol consumption |
| **≥0.80** | Rice or rice dishes; Salty snacks; Coke or other soda; Packaged or prepared food such as frozen dinners or take out; Non fast-food restaurants that serve typically non-Asian food (ex. American, Mexican, Italian restaurants) ; [Participant ethnicity] or other Asian style breakfast; [Participant ethnicity] or other Asian style dinner; Shop at [participant ethnicity] or other Asian food markets; American style supermarkets; Think about the "hot" and "cold" characteristics of foods; Asian food in diet |
| * Overall and individual measures of sampling adequacy range from between 0 and 1, with values >0.60 being satisfactory | |
