# Supplementary material for: Acculturating to multiculturalism: a new dimension of dietary acculturation among Asian American, Native Hawaiian, and Pacific Islander women in the San Francisco Bay Area, USA
Source: BMC Public Health. 2024 Aug 6;24:2128. doi: 10.1186/s12889-024-19435-4 (PMC11302078; doi:10.1186/s12889-024-19435-4)
Supplement: Supplementary file 2 — Supplementary Material 2 [file 12889_2024_19435_MOESM2_ESM.docx]

**Table III. Distribution of dietary acculturation factor by sociodemographic characteristics and acculturation among Asian American, Native Hawaiian, and Pacific Islander women (N=440)**

|  |  | **Asian Diet** | | | **Western Diet** | | | **Multicultural Diet** | | |
| --- | --- | --- | --- | --- | --- | --- | --- | --- | --- | --- |
| Characteristic | n | Not high | High | p-value | Not high | High | p-value | Not high | High | p-value |
| Age years |  |  |  | 0.002 |  |  | 0.001 |  |  | 0.002 |
| <50 | 205 | 51.0% | 37.7% |  | 41.2% | 57.5% |  | 48.6% | 42.5% |  |
| 50-59 | 131 | 30.3% | 28.8% |  | 30.6% | 28.1% |  | 32.7% | 24.0% |  |
| 60+ | 104 | 18.7% | 33.6% |  | 28.2% | 14.4% |  | 18.7% | 33.6% |  |
| Ethnicity |  |  |  | <0.001 |  |  | <0.001 |  |  | <0.001 |
| Chinese | 234 | 47.3% | 65.1% |  | 61.6% | 36.3% |  | 46.6% | 66.4% |  |
| Filipinx | 83 | 16.7% | 23.3% |  | 15.6% | 25.3% |  | 22.1% | 12.3% |  |
| NHPI | 8 | 2.4% | 0.7% |  | 0.7% | 4.1% |  | 1.7% | 2.1% |  |
| Other Asian American^a^ | 115 | 33.7% | 11.0% |  | 22.1% | 34.2% |  | 29.6% | 19.2% |  |
| Education |  |  |  | <0.001 |  |  | <0.001 |  |  | 0.29 |
| <High School | 75 | 6.8% | 37.7% |  | 21.1% | 8.9% |  | 16.7% | 17.8% |  |
| Some college or Associates | 91 | 18.0% | 26.0% |  | 21.8% | 18.5% |  | 22.1% | 17.8% |  |
| Bachelor’s degree | 165 | 42.5% | 27.4% |  | 31.0% | 50.7% |  | 38.8% | 34.9% |  |
| Graduate degree | 108 | 32.7% | 8.2% |  | 25.9% | 21.9% |  | 22.4% | 28.8% |  |
| Employment |  |  |  | 0.007 |  |  | 0.078 |  |  | 0.37 |
| Full-time | 190 | 48.0% | 33.6% |  | 41.5% | 46.6% |  | 44.9% | 39.7% |  |
| Part-time | 99 | 22.1% | 23.3% |  | 21.8% | 24.0% |  | 22.1% | 23.3% |  |
| Not working | 95 | 17.3% | 30.1% |  | 21.1% | 22.6% |  | 22.1% | 20.5% |  |
| Retired | 56 | 12.6% | 13.0% |  | 15.6% | 6.8% |  | 10.9% | 16.4% |  |
| Per capita household income to poverty ratio^b^ |  |  |  | <0.001 |  |  | 0.48 |  |  | 0.92 |
| <1.00 | 131 | 22.4% | 63.8% |  | 37.4% | 31.5% |  | 34.7% | 36.8% |  |
| 1.00-1.99 | 63 | 18.1% | 14.7% |  | 17.1% | 16.9% |  | 17.1% | 16.8% |  |
| 2.00+ | 176 | 59.4% | 21.6% |  | 45.5% | 51.6% |  | 48.2% | 46.4% |  |
| Marital Status |  |  |  | 0.004 |  |  | 0.37 |  |  | 0.045 |
| Married/living w partner | 291 | 63.3% | 71.9% |  | 67.3% | 63.7% |  | 62.2% | 74.0% |  |
| Formerly married | 67 | 13.9% | 17.8% |  | 16.0% | 13.7% |  | 15.6% | 14.4% |  |
| Single | 81 | 22.8% | 9.6% |  | 16.3% | 22.6% |  | 21.8% | 11.6% |  |
| Nativity |  |  |  | <0.001 |  |  | <0.001 |  |  | 0.13 |
| Foreign born | 289 | 51.4% | 94.5% |  | 72.8% | 51.4% |  | 63.3% | 70.5% |  |
| US born | 151 | 48.6% | 5.5% |  | 27.2% | 48.6% |  | 36.7% | 29.5% |  |
| Generational Status |  |  |  | <0.001 |  |  | <0.001 |  |  | 0.17 |
| Foreign born, age to US 10+ | 256 | 42.0% | 92.5% |  | 67.2% | 42.4% |  | 55.7% | 65.5% |  |
| Foreign born, age to US <10 | 33 | 10.4% | 2.1% |  | 6.6% | 9.7% |  | 8.7% | 5.5% |  |
| US born, 1st gen | 76 | 25.3% | 2.1% |  | 13.4% | 25.7% |  | 19.0% | 14.5% |  |
| US born, 2nd gen | 29 | 9.4% | 1.4% |  | 5.2% | 9.7% |  | 8.0% | 4.1% |  |
| US born, 3rd gen | 40 | 12.8% | 2.1% |  | 7.6% | 12.5% |  | 8.7% | 10.3% |  |
| English proficiency |  |  |  | <0.001 |  |  | <0.001 |  |  | 0.011 |
| Not good/Poor | 70 | 3.7% | 40.4% |  | 21.8% | 4.1% |  | 12.2% | 23.3% |  |
| Okay/Well | 119 | 19.4% | 42.5% |  | 29.6% | 21.9% |  | 27.6% | 26.0% |  |
| Very well | 59 | 16.7% | 6.8% |  | 10.9% | 18.5% |  | 12.6% | 15.1% |  |
| Only speaks English | 192 | 60.2% | 10.3% |  | 37.8% | 55.5% |  | 47.6% | 35.6% |  |

Notes:

“High” category represents the highest tertile and “not high” category represents the lower two tertiles.

**Only for those who reported being foreign-born.

^a^ Other Asian American groups included Japanese, Korean, South Asian, Southeast Asian, “Other Asian”, and Asian mixed ethnicity

^b^ Income/poverty ratio <1.00 indicates that family income was less than the poverty threshold; Income/poverty ratio between 1.00-1.99 indicates that family income was equal to or higher than poverty threshold; Income/poverty ratio 2.00+ generally indicates that the family is relatively well off.

**Table IV. Percentage distribution of each dietary acculturation factor by social characteristics, discrimination among Asian American, Native Hawaiian, and Pacific Islander women (n=440)**

|  |  | **Asian Diet** | | | **Western Diet** | | | **Multicultural Diet** | | |
| --- | --- | --- | --- | --- | --- | --- | --- | --- | --- | --- |
| Characteristic | n | Not high | High | p-value | Not high | High | p-value | Not high | High | p-value |
| Home ownership |  |  |  | <0.001 |  |  | 0.40 |  |  | 0.81 |
| Yes | 272 | 69.4% | 46.6% |  | 60.2% | 65.1% |  | 61.2% | 63.0% |  |
| No | 166 | 30.6% | 52.1% |  | 39.1% | 34.9% |  | 38.4% | 36.3% |  |
| Social network (# people) |  |  |  | <0.001 |  |  | 0.16 |  |  | 0.26 |
| 0-2 | 109 | 20.4% | 33.6% |  | 27.2% | 19.9% |  | 24.1% | 26.0% |  |
| 3-4 | 210 | 47.3% | 48.6% |  | 44.9% | 53.4% |  | 50.3% | 42.5% |  |
| ≥5 | 121 | 32.3% | 17.8% |  | 27.9% | 26.7% |  | 25.5% | 31.5% |  |
| Neighbourhood collective efficacy |  |  |  | 0.61 |  |  | 0.11 |  |  | 0.003 |
| Lower | 131 | 28.9% | 31.5% |  | 28.9% | 31.5% |  | 24.8% | 39.7% |  |
| Moderate | 137 | 32.7% | 28.1% |  | 34.4% | 24.7% |  | 31.6% | 30.1% |  |
| Higher | 172 | 38.4% | 40.4% |  | 36.7% | 43.8% |  | 43.5% | 30.1% |  |
| Experiences with discrimination1 |  |  |  | 0.26 |  |  | 0.031 |  |  | 0.85 |
| None | 102 | 20.4% | 24.0% |  | 20.4% | 24.0% |  | 20.7% | 23.3% |  |
| 1-2 | 91 | 19.4% | 19.9% |  | 21.8% | 15.1% |  | 19.0% | 20.5% |  |
| 3-4 | 152 | 31.0% | 35.6% |  | 35.0% | 27.4% |  | 33.7% | 30.1% |  |
| ≥5 | 129 | 29.3% | 20.5% |  | 22.8% | 33.6% |  | 26.5% | 26.0% |  |
| Perceived Stress Scale |  |  |  | 0.012 |  |  | 0.13 |  |  | 0.22 |
| Lower | 127 | 33.0% | 19.9% |  | 31.0% | 24.0% |  | 28.2% | 29.5% |  |
| Moderate | 143 | 31.6% | 34.2% |  | 29.6% | 38.4% |  | 30.3% | 37.0% |  |
| Higher | 175 | 35.4% | 45.9% |  | 39.5% | 37.7% |  | 41.5% | 33.6% |  |
| Reason for immigration2 (among foreign-born)** |  |  |  |  |  |  |  |  |  |  |
| Improve life | 188 | 34.7% | 58.9% | <0.001 | 45.6% | 37.0% | 0.086 | 44.2% | 39.7% | 0.37 |
| Better education | 138 | 24.8% | 44.5% | <0.001 | 35.0% | 24.0% | 0.019 | 33.0% | 28.1% | 0.30 |
| Join family | 130 | 19.4% | 50.0% | <0.001 | 31.6% | 25.3% | 0.17 | 27.9% | 32.9% | 0.28 |
| Find a job | 124 | 23.1% | 38.4% | <0.001 | 29.9% | 24.7% | 0.25 | 27.9% | 28.8% | 0.85 |
| Immigration-related stress (among foreign-born)** |  |  |  | 0.18 |  |  | 0.28 |  |  | 0.11 |
| Lower | 87 | 33.1% | 26.8% |  | 31.8% | 25.3% |  | 26.9% | 35.9% |  |
| Moderate | 84 | 31.1% | 26.8% |  | 26.6% | 36.0% |  | 28.0% | 31.1% |  |
| Higher | 118 | 35.8% | 46.4% |  | 41.6% | 38.7% |  | 45.2% | 33.0% |  |
| Social standing regarding money (among foreign-born)** |  |  |  | 0.13 |  |  | 0.56 |  |  | 0.37 |
| Better before | 85 | 33.3% | 45.8% |  | 39.8% | 42.2% |  | 40.5% | 40.0% |  |
| Same | 66 | 32.3% | 30.5% |  | 30.1% | 35.6% |  | 28.2% | 36.3% |  |
| Better in the US | 60 | 34.4% | 23.7% |  | 30.1% | 22.2% |  | 31.3% | 23.8% |  |
| Social standing regarding education (among foreign-born)** |  |  |  | 0.012 |  |  | 0.094 |  |  | 0.39 |
| Better before | 75 | 25.5% | 42.1% |  | 37.5% | 25.5% |  | 31.9% | 40.0% |  |
| Same | 81 | 38.3% | 37.2% |  | 33.9% | 51.1% |  | 40.7% | 32.5% |  |
| Better in the US | 59 | 36.2% | 20.7% |  | 28.6% | 23.4% |  | 27.4% | 27.5% |  |
| Social standing regarding job/occupation (among foreign-born)** |  |  |  | 0.097 |  |  | 0.10 |  |  | 0.88 |
| Better before | 91 | 44.2% | 52.5% |  | 50.0% | 44.4% |  | 47.5% | 50.8% |  |
| Same | 49 | 23.3% | 28.7% |  | 22.5% | 37.8% |  | 26.2% | 26.2% |  |
| Better in the US | 47 | 32.6% | 18.8% |  | 27.5% | 17.8% |  | 26.2% | 23.1% |  |

**Only for those who reported being foreign-born.

^1^ Each of the 8 items in the Experiences with Discrimination scale was coded as "ever experienced" (i.e., 0=never experienced; 1=rarely, sometimes, or often experienced) and summed to reflect the total number of experiences in lifetime. Thus, summed scores ranged from 0 (none of the situations experienced) to 8 (experienced all the discriminatory situations). To facilitate interpretation, the score was categorized into 0, 1-2, 3-4, or 5 or more discriminatory situations experienced.
