# Supplementary material for: Acculturating to multiculturalism: a new dimension of dietary acculturation among Asian American, Native Hawaiian, and Pacific Islander women in the San Francisco Bay Area, USA
Source: BMC Public Health. 2024 Aug 6;24:2128. doi: 10.1186/s12889-024-19435-4 (PMC11302078; doi:10.1186/s12889-024-19435-4)
Supplement: Supplementary file 3 — Supplementary Material 3 [file 12889_2024_19435_MOESM3_ESM.docx]

**Study ID: __________________**

**CONTROL**

**
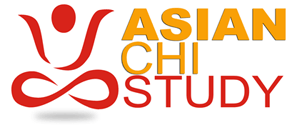
**

CONFIDENTIAL

Introduction

Thank you for participating in the Asian American Community Health Initiative Study. This survey will take about 30 minutes to fill out. The questions are about different topics such as: your living experience in the United States, physical activity, stress, smoking, alcohol, body measurement, etc.

We encourage you to try to answer all of the questions so that we can best understand your experiences, but you may skip any questions you do not wish to answer. Please know that all information that you give is confidential, will be shared only with authorized researchers and will only be used for research purposes. Individual information will not be linked to any specific person’s name; rather all information will be examined in a grouped way to protect your individual privacy. We want to emphasize that your participation is very important, and we thank you in advance for your participation.

Finally, when you are done, please mail back the survey in the self addressed stamped envelope. After completing and returning the survey, we will send you $15 for your time and effort taken to fill out the survey.

Thank you.

Instructions

###### PLEASE READ THESE INSTRUCTIONS CAREFULLY

- Answer each question as best you can.
- Put an **X** in the box next to your answer or circle your response, as instructions indicate. Please erase or cross out completely if you make any changes.

**Example: _1_  X** Yes

- Please follow any instructions that direct you to the next question**.**

**Example: _1_ X**  Never or rarely (**GO TO D39).**

- For a question with a line after it, please write the specific information on the line provided.

**Example: _1_ X**  What is it called: *Sunset District*____

- Mark only one response for each question, unless directed to **“CHECK ALL THAT APPLY.”** For those questions, please mark every response choice that applies to your situation.
- Please do your best to answer each question on your own. But if you needed help filling out the survey because of physical or visual problems, please let us know on the additional comments sheet at the end of this survey.
- Note that the enclosed Show Card #1 is to be used for the first 4 questions.
- A tape measure is provided in this packet for taking you body measurements (see Section II).

**Please refer to the picture in Show Card #1 to answer the following questions.**

**A1.** In the past 12 months, have you consumed any alcohol, including beer, wine,

champagne, sake, soju, liquor, mixed drinks, or any other type of alcohol?

❑ YES

❑ NO **(SKIP TO SECTION 2 PAGE 7)**

**A2.** In the past 12 months, how often did you usually drink beer?

Never or rarely ❑ (Go to **A4**)

1-3 times a MONTH ❑

**A3.** Which picture in Show Card #1 best represents the amount of beer you usually drank each time?

❑ A2 ❑ B2 ❑ C2 ❑ D2 ❑ E2 ❑ F2 ❑G2

1-3 times a WEEK ❑

4-6 times a WEEK ❑

Once a DAY ❑

2 or more times a DAY ❑

**A4.** In the past 12 months, how often did you usually drink **white**, **pink** or **rosé** wine,

champagne, sake, or soju?

Never or rarely ❑ (Go to **A6**)

**A5.** Which picture in Show Card #1, best represents the amount of white wine, pink or rosé wine, champagne, etc. you usually drank each time?

❑ A2 ❑ B2 ❑C2 ❑D2 ❑ E2 ❑F2 ❑ G2

1-3 times a MONTH ❑

1-3 times a WEEK ❑

4-6 times a WEEK ❑

Once a DAY ❑

2 or more times a DAY ❑

**A6.** In the past 12 months, how often did you usually drink **red** wine?

Never or rarely ❑ (Go to **A8**)

1-3 times a MONTH ❑

**A7** Which picture in Show Card #1, best represents the amount of red wine you usually drank each time?

❑ A2 ❑ B2 ❑ C2 ❑ D2 ❑ E2 ❑ F2 ❑ G2

1-3 times a WEEK ❑

4-6 times a WEEK ❑

Once a DAY ❑

2 or more times a DAY ❑

**A8.** In the past 12 months, how often did you usually drink liquor, such as whiskey or other liquor, mixed drinks or liqueurs?

Never or rarely ❑ (Go to **NEXT PAGE**)

1-3 times a MONTH ❑

**A9.** Which picture in Show Card #1, best represents the amount of liquor you usually drank each time? Please include **only** the liquor amount, **not** any soda or mixer you added to the liquor.

❑ A2 ❑ B2 ❑ C2 ❑ D2 ❑ E2 ❑ F2 ❑ G2

1-3 times a WEEK ❑

4-6 times a WEEK ❑

Once a DAY ❑

2 or more times a DAY ❑

Instructions for Taking Body Measurements

- All measurements should be done at the same time -- at least two hours after a meal.
- Wear only minimal clothing (such as underwear) or no clothing at all. Please do not wear a girdle or pantyhose.
- Measurements should be made while standing. A full-length mirror can help in positioning the tape. Or ask another person to help.
- Take two measurements of each body site and record them on the next page (to the nearest quarter inch). Measurements of the same site **may** differ slightly.
- Make sure the tape measure is horizontal and snug all the way around the body part without indenting the skin.
- Release the tape measure completely between each measurement.

Hip Measurements

The hip measurement should be taken at the largest point between your waist and thighs. When locating the correct spot, be sure to keep the tape measure horizontal.

Waist Measurements

Measure your "waist" at a point one inch above the naval ("belly button"), even if this is not your usual waistline.

Take measurement here

Take measurement here

**Record measurements here (to the nearest quarter inch,**

**For example, 32 1/4", 32 1/2", 32 3/4"):**

**B1.** FIRST WAIST MEASUREMENT:______

**B2**. FIRST HIP MEASUREMENT:_______

**B3.** SECOND WAIST MEASUREMENT: ______

**B4.** SECOND HIP MEASUREMENT: _______

**If you were born in the United States, SKIP TO Section 4 on page 16.**

**If you were NOT born in the United States, please answer the following questions:**

**I1.** The following are a list of reasons that people give for coming to the United States. Please check the box below the response indicating how important each one of these reasons was for you and/or your family to come to the US.

|  | **Not important** | **Somewhat important** | **Very important** | **Does not apply** |
| --- | --- | --- | --- | --- |
| a. **To find employment or a job** |  |  |  |  |
| b. **To improve your life or that of your family and look for better opportunities** |  |  |  |  |
| c. **To join other family members already living in the US** |  |  |  |  |
| d. **Because of the political situation in your country of origin** |  |  |  |  |
| e. **Because you or your family were mistreated for political reasons** |  |  |  |  |
| f. **For medical care** |  |  |  |  |
| g. **To get a better education** |  |  |  |  |
| h. **Because of marital or family problems** |  |  |  |  |

**I2.** Using the ladders below, please circle the number that corresponds to where you feel you currently stand compared to other people in the United States in terms of your **money.**

**Ladder A - MONEY**

**(CIRCLE 1-10)**

10 = The people who are the best off –

those who have the most money.

10

9

8

7

6

5

4

3

2

1

1 = The people who are the worst off –

those who have the least money.

**I3.** Using the ladders below, please circle the number that corresponds

to where you feel you currently stand compared to other people in

the United States in terms of your **education.**

**Ladder B - EDUCATION**

**(CIRCLE 1-10)**

10 = The people who are the best off – those who have the most education.

10

9

8

7

6

5

4

3

2

1

1 = The people who are the worst off –

those who have the least education.

**I4.** Using the ladders below, please circle the number that corresponds

to where you feel you currently stand compared to other people in

the United States in terms of your **job or occupation.**

**Ladder C – JOB or OCCUPATION**

**(CIRCLE 1-10)**

10 = The people who are the best off – those who have the most respected job or occupation .

1 = The people who are the worst off –

those who have the least respected job or occupation .

10

9

8

7

6

5

4

3

2

1

**If you were 20 years old or YOUNGER when you first came to the United States to live, SKIP THIS SECTION AND GO TO Section 4 on page 16.**

**If you were 21 years old or OLDER answer the following questions:**

**I5.** Where did you stand **BEFORE** you came to the US to live in terms of your **money.**

**Ladder A - MONEY**

**(CIRCLE 1-10)**

10 = The people who are the best off –

those who have the most money.

10

9

8

7

6

5

4

3

2

1

1 = The people who are the worst off –

those who have the least money.

**I6.** Where did you stand **BEFORE** you came to the US to live in terms of your

**education**.

**Ladder B - EDUCATION**

**(CIRCLE 1-10)**

10 = The people who are the best off – those who have the most education.

10

9

8

7

6

5

4

3

2

1

1 = The people who are the worst off

those who have the least education.

**I7.** Where did you stand **BEFORE** you came to the US to live in terms of your

**job or occupation.**

**Ladder C – JOB or OCCUPATION**

**(CIRCLE 1-10)**

10 = The people who are the best off – those who have the most respected job or occupation.

10

9

8

7

6

5

4

3

2

1

1 = The people who are the worst off –

those who have the least respected job or occupation.

**S1.** The next questions are about the overall stress you may have felt

during the past 12 months.

| ***During the past 12 months, how often…*** | | **Never** | **Almost Never** | **Sometimes** | **Fairly Often** | **Very**  **Often** |
| --- | --- | --- | --- | --- | --- | --- |
| a. | **were you upset because of something that happened unexpectedly?** |  |  |  |  |  |
| b. | **did you feel that you were unable to control the important things in your life?** |  |  |  |  |  |
| c. | **did you feel "stressed"?** |  |  |  |  |  |
| d. | **did you feel confident about your ability to handle your personal problems?** |  |  |  |  |  |
| e. | **did you feel that things were going your way?** |  |  |  |  |  |
| f. | **did you feel that you could not cope with all the things that you had to do?** |  |  |  |  |  |
| g. | **were you able to control irritations in your life?** |  |  |  |  |  |
| h. | **did you feel that you were on top of things?** |  |  |  |  |  |
| i. | **were you angered because of things that were outside of your control?** |  |  |  |  |  |
| j. | **did you feel difficulties were piling up so high that you could not overcome them?** |  |  |  |  |  |

**S2.** I will now ask you some questions about how you see yourself. Please tell me how much you agree with each statement.

| Check one response for each statement below: | | **Strongly Agree** | **Agree** | **Disagree** | **Strongly Disagree** |
| --- | --- | --- | --- | --- | --- |
| a. | **You’ve always felt that you could make of your life pretty much what you wanted to make of it.** |  |  |  |  |
| b. | **Once you make up your mind to do something, you stay with it until the job is completely done.** |  |  |  |  |
| c. | **You like doing things that other people thought could not be done.** |  |  |  |  |
| d. | **When things don’t go the way you want them to, that just makes you work even harder.** |  |  |  |  |
| e. | **Sometimes, you feel that if anything is going to be done right, you have to do it yourself.** |  |  |  |  |
| f. | **It’s not always easy, but you manage to find a way to do the things you really need to get done.** |  |  |  |  |
| g. | **Very seldom have you been disappointed by the results of your hard work.** |  |  |  |  |
| h. | **You feel you are the kind of individual who stands up for what she believes in, *regardless of the consequences.*** |  |  |  |  |
| i. | **In the past, even when things got really tough, you never lost sight of your goals.** |  |  |  |  |
| j. | **It’s important for you to be able to do things the way you want to do them rather than the way other people want you to do them.** |  |  |  |  |
| k. | **You don’t let your personal feelings get in the way of doing a job.** |  |  |  |  |
| l. | **Hard work has really helped you to get ahead in life.** |  |  |  |  |

The following questions are about your usual sleep patterns **over the past 12 months**.

**SL1.** During a typical 24-hour period, how many hours of actual sleep did you usually get? (This may be different than the number of hours you spent in bed.)

____ Hours

**SL2.** Over the past 12 months, what time did you **usually** go to bed?

- before 9:00 PM
- between 9:00 – 9:59 PM
- between 10:00 – 10:59 PM
- between 11:00 – 11:59 PM
- after 12:00 AM

**SL3.** Over the past 12 months, how long did it usually take you to fall asleep each night?

- <15 minutes
- 16-30 minutes
- 31-60 minutes
- more than 60 minutes

**SM1.** Have you smoked at least 100 cigarettes over your entire lifetime?

❑ NO (GO TO **SM6 PAGE 20**)

❑ YES (GO TO **SM2**)

**SM2.** How old were you when you first smoked fairly regularly?

|__|__| YRS OLD (GO TO **SM3**)

❑ NEVER SMOKED REGULARY (GO TO **SM6 PAGE 20**)

**SM3.** Do you currently smoke?

❑ NO (GO TO **SM6**)

❑ YES

**SM4.** How old were you when you last smoked fairly regularly?

|__|__| YRS OLD

**SM5.** On average, about how many cigarettes a day do/did you smoke?

|__|__| CIGARETTES PER DAY

1 pack = 20 cigarettes

|__| PACKS PER DAY

❑ DON'T KNOW

**SM6.** When you were under age 18, did you live with anyone who smoked cigarettes in the house?

❑ NO (GO TO **SM8**)

❑ YES (GO TO **SM7**)

**SM7.** How many people in the household smoked? |__|__|

**SM8.** During the past 10 years, did you live with anyone who smoked cigarettes?

❑ NO (GO TO **SM12 PAGE 21**)

❑ YES (GO TO **SM9**)

**SM9.** For how many years (during the past 10 years) did you live with someone who smoked?

|__|__| YRS

**SM10.** How many hours a day were you usually exposed to their cigarette smoke?

|__|__| HRS PER DAY

**SM11.** On average, how smoky was the house?

❑ Very smoky

❑ Fairly smoky

❑ A little smoky

**SM12.** During the past 10 years, have you **regularly** been exposed to cigarette smoke in places outside your home (for example, at work, with friends, during commuting, or in other social situations) **for 2 or more hours a week**?

❑ NO (GO TO **SECTION 7, PAGE 23**)

❑ YES (GO TO **SM13**)

**SM13.** For how many years (during the past 10 years) have you been exposed to smoke in these social settings?

|__|__| YRS

**SM14.** For how many hours a week were you usually exposed to cigarette smoke in these social settings?

|__|__| HOURS PER WEEK

**SM15.** On average, how smoky were these places?

❑ Very smoky

❑ Fairly smoky

❑ A little smoky

The next questions are about physical activity. Please think about recreational activities, activity on the job, and activity around the house. For example: household chores.

**P1.** The first set of questions refers to **STRENUOUS** physical activities, such as vacuuming, washing windows, heavy lifting, farm work, mowing the lawn, swimming laps, aerobics, running, basketball, riding a bike on hills, or racquetball. For each of the time periods below, please indicate how much time and for how many months of the year you did these types of activities.

| **Time period** | **How many HOURS A WEEK did you spend doing strenuous activities?** | **In how many MONTHS DURING THE YEAR did you do strenuous activities?** |
| --- | --- | --- |
| During high school (age 15 – 18) | - None ❑ 3 hours - ½ hour ❑ 4-6 hours - 1 hour ❑ 7-10 hours - 1 ½ hours ❑ 11 or more hours - 2 hours ❑ Not applicable | - 1-3 months - 4-6 months - 7-9 months - 10-12 months |
| Between the ages of 20 and 35 | - None ❑ 3 hours - ½ hour ❑ 4-6 hours - 1 hour ❑ 7-10 hours - 1 ½ hours ❑ 11 or more hours - 2 hours ❑ Not applicable | - 1-3 months - 4-6 months - 7-9 months - 10-12 months |
| **Time period** | **How many HOURS A WEEK did you spend doing strenuous activities?** | **In how many MONTHS DURING THE YEAR did you do strenuous activities?** |
| Between the ages of 45 and 55 | - None ❑ 3 hours - ½ hour ❑ 4-6 hours - 1 hour ❑ 7-10 hours - 1 ½ hours ❑ 11 or more hours - 2 hours ❑ Not applicable | - 1-3 months - 4-6 months - 7-9 months - 10-12 months |
| During the past 12 months | - None ❑ 3 hours - ½ hour ❑ 4-6 hours - 1 hour ❑ 7-10 hours - 1 ½ hours ❑ 11 or more hours - 2 hours ❑ Not applicable | - 1-3 months - 4-6 months - 7-9 months - 10-12 months |

**P2.** The next set of questions refers to **MODERATE** physical activities, such as brisk walking, walking to school or work, shopping, running errands, golf, volleyball, riding a bike on level streets, recreational tennis, or softball.

| **Time period** | **How many HOURS A WEEK did you spend doing moderate activities?** | **In how many MONTHS OF THE YEAR did you do moderate activities?** |
| --- | --- | --- |
| During high school (age 15-18) | - None ❑ 3 hours - ½ hour ❑ 4-6 hours - 1 hour ❑ 7-10 hours - 1 ½ hours ❑ 11 or more hours - 2 hours ❑ Not applicable | - 1-3 months - 4-6 months - 7-9 months - 10-12 months |
| Between the ages of 20 and 35 | - None ❑ 3 hours - ½ hour ❑ 4-6 hours - 1 hour ❑ 7-10 hours - 1 ½ hours ❑ 11 or more hours - 2 hours ❑ Not applicable | - 1-3 months - 4-6 months - 7-9 months - 10-12 months |
| Between the ages of 45 and 55 | - None ❑ 3 hours - ½ hour ❑ 4-6 hours - 1 hour ❑ 7-10 hours - 1 ½ hours ❑ 11 or more hours - 2 hours ❑ Not applicable | - 1-3 months - 4-6 months - 7-9 months - 10-12 months |

| **Time period** | **How many HOURS A WEEK did you spend doing moderate activities?** | **In how many MONTHS OF THE YEAR did you do moderate activities?** |
| --- | --- | --- |
| During the past 12 months | - None ❑ 3 hours - ½ hour ❑ 4-6 hours - 1 hour ❑ 7-10 hours - 1 ½ hours ❑ 11 or more hours - 2 hours ❑ Not applicable | - 1-3 months - 4-6 months - 7-9 months - 10-12 months |

**P3.** During the past 12 months, how many **hours a week** did you usually spend doing the

following activities?

| Standing or walking around at work or away from home | **Hours per week** |
| --- | --- |
|  | - 0-1 ❑ 4-6 ❑ 11-20 ❑ 31-39 - 2-3 ❑ 7-10 ❑ 21-30 ❑ 40+ |
| Standing or walking around at home | - 0-1 ❑ 4-6 ❑ 11-20 ❑ 31-39 - 2-3 ❑ 7-10 ❑ 21-30 ❑ 40+ |
| Sitting at work | ❑0-1 ❑ 4-6 ❑ 11-20 ❑ 31-39   - 2-3 ❑ 7-10 ❑ 21-30 ❑ 40+ - not applicable, not working |
| Sitting or driving in a car, bus, or train | - 0-1 ❑ 4-6 ❑ 11-20 ❑ 31-39 - 2-3 ❑ 7-10 ❑ 21-30 ❑ 40+ |
| Sitting or reclining while watching TV | - 0-1 ❑ 4-6 ❑ 11-20 ❑ 31-39 - 2-3 ❑ 7-10 ❑ 21-30 ❑ 40+ |
| Sitting or reclining while reading | - 0-1 ❑ 4-6 ❑ 11-20 ❑ 31-39 - 2-3 ❑ 7-10 ❑ 21-30 ❑ 40+ |
| Sitting for other reasons (e.g., playing games, working at a desk, sewing) | - 0-1 ❑ 4-6 ❑ 11-20 ❑ 31-39 - 2-3 ❑ 7-10 ❑ 21-30 ❑ 40+ |

**P4.** How many flights of stairs (not individual steps but going from one floor to another) do you climb each day?

- None
- 1-2
- 3-4
- 5-9
- 10-14
- 15 or more

**P5.** What is your normal walking pace?

- Slow (less than 2 miles per hour)
- Normal (2-2.9 miles per hour)
- Brisk (3-3.9 miles per hour)
- Very brisk (4 miles per hour or faster)
- Unable to walk

The following questions are about your current neighborhood.

**N1.** In what year did you first move to your current address? ____________

*Year*

**We would like you to define the area you consider your neighborhood.**

A neighborhood is a section of a city or town. Examples of neighborhoods

include the Sunset District, Chinatown, Rockridge, etc.

**N2.** Does your neighborhood have a name?

- Yes → GO TO **N3**
- No → GO TO **N4**
- Don’t know → GO TO **N4**

**N3.** What is it called?________________________________________

**N4.** How often do you feel safe in your current neighborhood?

*[Check one]*

| **None of the time** | **Some of the time** | **Most of the time** | **All of the time** |
| --- | --- | --- | --- |
| 🞏 | 🞏 | 🞏 | 🞏 |

**N5.** Thinking about your neighborhood as a whole, please indicate if the following issues are a problem

*[check one box for each issue listed below]:*

|  | **Not really a problem** | **Minor problem** | **Somewhat serious problem** | **Very serious problem** |
| --- | --- | --- | --- | --- |
| Crime in your neighborhood | 🞏 | 🞏 | 🞏 | 🞏 |
| Traffic | 🞏 | 🞏 | 🞏 | 🞏 |
| A lot of noise | 🞏 | 🞏 | 🞏 | 🞏 |
| Trash and litter | 🞏 | 🞏 | 🞏 | 🞏 |
| Lighting at night | 🞏 | 🞏 | 🞏 | 🞏 |

**N6.** The next questions are about your neighbors:

*[check one box for each question below]:*

|  | **Often** | **Sometimes** | | **Rarely** | **Never** |
| --- | --- | --- | --- | --- | --- |
| 1. How often do you see neighbors talking outside in the yard, on the street, at the corner park, etc? | 🞏 | 🞏 | 🞏 | | 🞏 |
| 1. How often do neighbors watch out for each other, such as calling if they see a problem? | 🞏 | 🞏 | 🞏 | | 🞏 |
|  | **A lot** | **Some** | | **Few** | **None** |
| 1. How many neighbors do you know by name? | 🞏 | 🞏 | | 🞏 | 🞏 |
| 1. How many neighbors do you have a friendly talk with at least once a week? | 🞏 | 🞏 | | 🞏 | 🞏 |
| e. How many neighbors could you call on for assistance in doing something around your home or yard or to “borrow a cup of sugar ” or some other small favor? | 🞏 | 🞏 | | 🞏 | 🞏 |

**N7.** Are there any groups in your neighborhood such as community associations, social clubs, book clubs, churches/spiritual centers, or faith-based organizations?

- Yes→ GO TO **N8**
- No → GO TO **H1 PAGE 32**
- Don’t know → GO TO **H1 PAGE 32**

**N8.** Are you actively involved in any of these groups?

- Yes
- No

The next several questions are about your doctors and other healthcare providers.

**H1.** Over the past 12 months, did you have one person that you think of as your primary care provider/ personal doctor?

- Yes → GO TO **H2**
- No → GO TO **H4 PAGE 33**

**H2.** Why did you choose this person as your personal doctor?

(**Check all that apply**)

- - My insurance/ health coverage allows me to see this particular doctor/ practice
  - His/Her office is close by to where I live or easy for me to get to
  - My doctor speaks the same language as me
  - I like the way s/he treats me
  - It is easy to get an appointment with my doctor
  - I was referred by someone
  - Other________

**H3.** Is this doctor of the same race/ethnicity as you?

- Yes
- No

**H4.** If you could choose, would you prefer to be treated by doctors of your own racial/ethnic group, another racial/ethnic group, or do you NOT have a preference?

Same racial/ethnic group

Another racial/ethnic group

No preference

**H5**. In general, how concerned are you that you may be treated unfairly *because* of your race/ethnicity when seeking medical care?

|  | - Not at all concerned |
| --- | --- |
|  | - A little concerned |
|  | - Somewhat concerned |
|  | - Extremely concerned |

**H6.** In general, how concerned are you that people of your same race/ethnicity may be treated unfairly *because* of their race/ethnicity when seeking medical care? This could include a family member, friend, or someone you do not know personally.

|  | - Not at all concerned |
| --- | --- |
|  | - A little concerned |
|  | - Somewhat concerned |
|  | - Extremely concerned |

**Please provide any additional comments regarding this survey, if you wish:**

*Thank you for completing this survey!*

Please return this completed booklet in the enclosed postage-paid return envelope.
