# Supplementary material for: Acculturating to multiculturalism: a new dimension of dietary acculturation among Asian American, Native Hawaiian, and Pacific Islander women in the San Francisco Bay Area, USA
Source: BMC Public Health. 2024 Aug 6;24:2128. doi: 10.1186/s12889-024-19435-4 (PMC11302078; doi:10.1186/s12889-024-19435-4)
Supplement: Supplementary file 4 — Supplementary Material 4 [file 12889_2024_19435_MOESM4_ESM.docx]

**Asian American CHI immigrant study**

**Survey for controls**

DATE: ____________________ TIME START:

STUDY ID: ________________ TIME END:

INTERVIEWER:_____________________

INSTRUCTIONS TO INTERVIEWER:

Read all **Bold** black type. The questions are in **BOLD** black type, as well as some instructions and introductions to various sections. Do not read choices that are in ALL CAPS. The choice “I don’t know” is indicated by “D/K”, and if the participant refuses to answer a questions, select “REFUSED.” Do not give “D/K” as an answer choice but, if, after probing, the participant says that they do not know, you would then circle that choice. Instructions in **BLUE BOLD** fonts are either Instructions for the Interviewer or optional, additional explanations or probes you can read to the participant—they are not required to be read. The fonts in red are skip patterns to be followed. *The fonts in italics are instructions to the interviewers.*

READ THESE INSTRUCTIONS TO PARTICIPANT:

**Thank you for agreeing to participate in the Asian American Community Health Initiative Study. During this interviewer, we will be asking you a variety of questions. You may skip any questions that you do not feel comfortable answering. You may also stop answering questions at any time. You can decide not to participate simply by telling me that you do not want to answer any questions at all.**

**We appreciate your time and effort in helping us with this research study.**

**Do you have any questions before we begin?**

1. Yes→ answer as appropriate
2. No→begin below with Diet and dietary acculturation Section

SECTION: DIET AND DIETARY ACCULTURATION

1. **We would like to gather some basic information about you. What is your date of birth?**

Month _____ Day ____ Year ____

D/K

REFUSED

1. **How old are you? _____**

**The next set of questions refers to your usual diet in the past 12 months. Please think about what you ate at home and in restaurants, including meals and snacks. I will ask you how often you ate certain foods or drank certain beverages. Please refer to Show Card #1 for frequency categories to help you answer these questions. For some foods, I will also ask you how much you usually ate. Please refer to the pictures on Show Card #2 to help you answer these questions.**

Show Card #1

Frequency categories

never or rarely

1 to 3 times a MONTH (that is LESS than once a week)

1 to 3 times a WEEK

4 to 6 times a WEEK

once a DAY

2 or more times a DAY

Show Card #2 - Picture of serving sizes

| FOOD/BEVERAGE | **How often did you usually (eat/drink) (FOOD/BEVERAGE) in the past 12 months?** | | | | | |  |  |
| --- | --- | --- | --- | --- | --- | --- | --- | --- |
|  | **never / rarely** | **1-3/mo** | **1-3/wk** | **4-6/wk** | **1/d** | **2+/d** | DK | REF |
| **D1. Fruit** |  |  |  |  |  |  |  |  |
| **D2. Dried apricots or dates** |  |  |  |  |  |  |  |  |
|  | | | **D3. Which picture on Show Card #2 best represents the amount of dried apricots or dates you usually ate each time?**  **\|__\| A \|__\| B \|__\| C \|__\| D** | | | |  |  |
| **D4. Vegetables (not counting potatoes or light green lettuce)** |  |  |  |  |  |  |  |  |
| **D5. Edamame or soybeans** |  |  |  |  |  |  |  |  |
|  | | | **D6. Which picture on Show Card #2 best represents the amount of edamame or soybeans you usually ate each time?**  **\|__\| A \|__\| B \|__\| C \|__\| D** | | | |  |  |
| **D7. Cheese** |  |  |  |  |  |  |  |  |
| **D8. Tofu** |  |  |  |  |  |  |  |  |
|  | | | **D9. Which picture on Show Card #2 best represents the amount of tofu you usually ate each time?**  **\|__\| A \|__\| B \|__\| C \|__\| D** | | | |  |  |
| **D10. Rice or rice dishes** |  |  |  |  |  |  |  |  |
| **D11. Ground beef or hamburgers** |  |  |  |  |  |  |  |  |
| **D12. Fish or fish stew** |  |  |  |  |  |  |  |  |
| **D13. Pizza or western-style pasta, including spaghetti and lasagna** |  |  |  |  |  |  |  |  |
| **D14. Western-style bread, rolls, or bagels** |  |  |  |  |  |  |  |  |
| **D15. Asian-style bread, such as pan de sal and naan** |  |  |  |  |  |  |  |  |
| **D16. Cereal** |  |  |  |  |  |  |  |  |
| **D17. Soy nuts** |  |  |  |  |  |  |  |  |
|  | | | **D18. Which picture on Show Card #2 best represents the amount of soynuts you usually ate each time?**  **\|__\| A \|__\| B \|__\| C \|__\| D** | | | |  |  |
| **D19. Protein or "power" bars** |  |  |  |  |  |  |  |  |
|  |  |  | **D20. Do the protein or power bars you eat contain soy?**  \|__\| YES \|__\| NO \|__\| SOMETIMES \|__\| DON'T KNOW | | | |  |  |
| **D21. Salty snacks (eg.potato chips, tortilla chips, pretzels)** |  |  |  |  |  |  |  |  |
| **D22. Black licorice** |  |  |  |  |  |  |  |  |
| **D23. Chocolate or other candy** |  |  |  |  |  |  |  |  |
| **D24. Doughnuts** |  |  |  |  |  |  |  |  |
| **D25. Cake, pie, or cookies** |  |  |  |  |  |  |  |  |
| **D26. Butter or margarine** |  |  |  |  |  |  |  |  |
| **BEVERAGES** | | | | | | |  |  |
| **D27. Soy milk** |  |  |  |  |  |  |  |  |
|  | | | **D28. Which picture on Show Card #2 best represents the amount of soymilk you usually drank each time?**  **\|__\| A2 \|__\| B2 \|__\| C2 \|__\| D2 \|__\| E2 \|__\| F2 \|__\| G2** | | | |  |  |
| **D29. Whole milk, 2%, 1%, or skim milk** |  |  |  |  |  |  |  |  |
| **D30. Coke or other soda** |  |  |  |  |  |  |  |  |
|  | | | **D31. Which picture on Show Card #2 best represents the amount of coke or soda you usually drank each time?**  **\|__\| A2 \|__\| B2 \|__\| C2 \|__\| D2 \|__\| E2 \|__\| F2 \|__\| G2** | | | |  |  |
| **D32. Tea** |  |  |  |  |  |  |  |  |
| **D33. Fruit juices** |  |  |  |  |  |  |  |  |

**Still referring to the past 12 months and using Show Card #1.**

| **How often did you eat:** | **Never / rarely** | **1-3/mo** | **1-3/wk** | **4-6/wk** | **1/d** | **2+/d** | Don't know | REF |
| --- | --- | --- | --- | --- | --- | --- | --- | --- |
| **D34. Homemade food or food that was prepared in your home?** |  |  |  |  |  |  |  |  |
| **D35. packaged or prepared foods such as frozen dinners or take out.** |  |  |  |  |  |  |  |  |
| **D36. at fast-food restaurants, that typically serve non-Asian food, such as McDonalds, Subway, or Dominos Pizza** |  |  |  |  |  |  |  |  |
| **D37. at other types of non fast-food restaurants serving typically non-Asian food (ex. American, Mexican, Italian restaurants)?** |  |  |  |  |  |  |  |  |

**Now please refer to the frequency categories on Show Card #3 to help you answer the following questions.**

Show Card #3

Frequency categories

Never or rarely

Sometimes

Frequently

| **How often did you:** | **never / rarely** | **sometimes** | **frequently** | D/K | REF |
| --- | --- | --- | --- | --- | --- |
| **D38. eat foods that are fermented, pickled, or traditionally preserved in other ways (such as fish sauce, kimchi, Japanese pickled vegetables, salted or preserved eggs, preserved tofu, salted fish, or Chinese sausage (lap chong)?** |  |  |  |  |  |
| **D39.** **eat a** *[participant ETHNICITY]* **or other Asian-style breakfast?** | \|__\| **don't eat breakfast**  **\|__\| eat breakfast but not an Asian-style one** |  |  |  |  |
| **D40**. **eat a *[participant ETHNICITY]* or other Asian-style dinner?** |  |  |  |  |  |
| **D41.** **shop at** *[participant ETHNICITY]* **or other Asian food markets?** |  |  |  |  |  |
| **D42. shop at American-style supermarkets?** |  |  |  |  |  |

**In some Asian cultures the balance of foods with "hot" and "cold" or "yin" and "yang" properties is part of tradition.**

**D43. How often do you think about the "hot" and "cold" characteristics of foods?"**

**|__| never/rarely** (GO TO D45) **|__| sometimes |__| frequently** |__| don't know

|__| N/A – Not part of ppt’s Asian culture / q does not make sense to ppt. (if D/K, N/A or REF skip to D45)

|__| REF

**D44**. **How often do you use these “hot” and “cold” characteristics to decide what to eat?**

**|__| never/rarely** **|__| sometimes |__| frequently**  |__| don't know

**|__| REF**

**D45. During the past 12 months, did your diet include** (READ OPTIONS) ...

**|__| mostly Asian foods**

**|__| more Asian than non-Asian foods**

**|__| an equal amount of Asian and non-Asian foods**

**|__| more non-Asian foods than Asian foods**

**|__| mostly non-Asian foods**

|__| DON'T KNOW

**|__|** REFUSED

**The last set of diet questions refer to your usual diet when you were about 12 years old.**

| FOOD/BEVERAGE | **How often did you usually** (eat/drink) (FOOD/BEVERAGE) **when you were about 12 years old**? | | | | | | | |
| --- | --- | --- | --- | --- | --- | --- | --- | --- |
|  | **never / rarely** | **1-3/mo** | **1-3/wk** | **4-6/wk** | **1/d** | **2+/d** | Don't know | Ref |
| **D46. Dried apricots or dates** |  |  |  |  |  |  |  |  |
| **D47. Edamame or soybeans** |  |  |  |  |  |  |  |  |
| **D48. Tofu** |  |  |  |  |  |  |  |  |
| **D49. Soy nuts** |  |  |  |  |  |  |  |  |
| **D50. Protein or "power" bars made with soy** |  |  |  |  |  |  |  |  |
| **D51. Black licorice** |  |  |  |  |  |  |  |  |
| **D52. Doughnuts** |  |  |  |  |  |  |  |  |
| **D53. Soy milk** |  |  |  |  |  |  |  |  |

SECTION: BODY SIZE

**The next set of questions are about your height and weight at different times in your life.**

**B1. How much did you usually weigh over the past 12 months?** |__|__|__| POUNDS 1

KGS2

**|__|** D/K

**|__|** REFUSED

**B2. How tall were you between the ages of 30 and 35?**

|__| FEET |__|__| INCHES OR |__|__|__| CENTIMETERS

**|__|** D/K

**|__|** REFUSED

B3-B5. **Excluding times when you may have been pregnant, what was your usual weight when you were age** ...

B3. 18 |__|__|__| POUNDS 1 KGS 2 **|__|** D/K **|__|** REFUSED

B4. 30-35 |__|__|__| POUNDS 1 KGS 2 |__|n/a (age 29 or under) **|__|** D/K **|__|** REFUSED

B5. 50-55 |__|__|__| POUNDS 1 KGS 2 |__|n/a (age 49 or under) **|__|** D/K **|__|** REFUSED

**B6. Please look at Show Card #4. Which picture on Show Card #4 best represents your body shape at age 12? |__| |__|** D/K **|__|** REFUSED

Show Card #4:

******

SECTION: SOCIAL NETWORKS

**We are now going to move on to questions about relationships you may have with different people in your life.**

**Important Matters**

S1. **From time to time, people discuss IMPORTANT MATTERS with other people, and have people they depend on for help. These can be family, friends, or others who have been really helpful to you. What we are interested in are the people whom you are most likely to talk to about really important matters in your life, regardless of whether these people live near you or far away.**

**Over the past 12 months, who have been the people in your life with whom you discuss important matters? Who are the people you can really count on? These can be people that you see, talk on the phone or via the computer, email, text, or IM (instant message) with.**

**Please tell me their initials or first names.**

**[Record names in column (1) on network form. For each name, ask the following questions.]**

**S2. Is ___ male or female?**

**S3. How old is ____? Your best guess is fine. |__|** D/K **|__|** REFUSED

**S4. To your best knowledge, what racial/ethnic group does ________belong to?**

1. **Asian (please specify: __________)**
2. **non-Hispanic White**
3. **Hispanic**
4. **African American**
5. **Other (please specify: ___________)**
6. D/K
7. REFUSED

**S5. Please look at Show Card #5, how are you connected to ____?**

**|__|** D/K **|__|** REFUSED

**[Record all that apply]**

**S6. How close are you to ______? Would you say very close, sort of close, or not very close?**

1 = Very close 2 = Sort of close 3 = Not very close

**|__|** D/K **|__|** REFUSED

S7. **How often do you interact with ____? Include times you see, talk, email, text, or IM (instant message) with (him/her). Would you say often, occasionally, or hardly ever? |__|** D/K **|__|** REFUSED

1 = Often 2 = Occasionally 3 = Hardly ever

| **1)**  **Full Name, Initials, Other Identifiers** | **2)**  **Gender** | **3)**  **Age** | **4)**  **Race/ethnicity** | **5)**  **Connection** | **6)**  **How Close** | **7)**  **Contact** | D/K | REFUSED |
| --- | --- | --- | --- | --- | --- | --- | --- | --- |
| #1 | M F |  |  |  | 1 2 3 | 1 2 3 |  |  |
| #2 | M F |  |  |  | 1 2 3 | 1 2 3 |  |  |
| #3 | M F |  |  |  | 1 2 3 | 1 2 3 |  |  |
| #4 | M F |  |  |  | 1 2 3 | 1 2 3 |  |  |
| #5 | M F |  |  |  | 1 2 3 | 1 2 3 |  |  |
| #6 | M F |  |  |  | 1 2 3 | 1 2 3 |  |  |
| #7 | M F |  |  |  | 1 2 3 | 1 2 3 |  |  |
| #8 | M F |  |  |  | 1 2 3 | 1 2 3 |  |  |
| #9 | M F |  |  |  | 1 2 3 | 1 2 3 |  |  |
| #10 | M F |  |  |  | 1 2 3 | 1 2 3 |  |  |
| #11 | M F |  |  |  | 1 2 3 | 1 2 3 |  |  |
| #12 | M F |  |  |  | 1 2 3 | 1 2 3 |  |  |
| #13 | M F |  |  |  | 1 2 3 | 1 2 3 |  |  |
| #14 | M F |  |  |  | 1 2 3 | 1 2 3 |  |  |
| #15 | M F |  |  |  | 1 2 3 | 1 2 3 |  |  |
| #16 | M F |  |  |  | 1 2 3 | 1 2 3 |  |  |
| #17 | M F |  |  |  | 1 2 3 | 1 2 3 |  |  |
| #18 | M F |  |  |  | 1 2 3 | 1 2 3 |  |  |
| #19 | M F |  |  |  | 1 2 3 | 1 2 3 |  |  |
| #20 | M F |  |  |  | 1 2 3 | 1 2 3 |  |  |

Density Matrix

**► USE MATRIX FORM, FILL IN NAMES:**

**We would now like you to think about the relationships between these people.**

**Some of them may be total strangers in the sense that they wouldn’t recognize one another if they bumped into each other on the street.**

**Others may know each other a bit, or they may be especially close.**

**First, think about** (1 ) and (2 ).

**How close are they to one another**: 1 = **Very close**

2 **= Sort of close**

3 **= Not very close**

4 **= Don’t know each other**

8 **=** D/K

9= REFUSED

|  | ______  **2** | **______**  **3** | **______**  **4** | **______**  **5** | **_______**  **6** | | **_______**  **7** | **_______**  **8** | **_______**  **9** | **_______**  **10** |
| --- | --- | --- | --- | --- | --- | --- | --- | --- | --- | --- |
| **1 _______** | ______ | ______ | ______ | ______ | | ______ | ______ | ______ | ______ | ______ |
| **2 _______** |  | ______ | ______ | ______ | | ______ | ______ | ______ | ______ | ______ |
| **3 _______** |  |  | ______ | ______ | | ______ | ______ | ______ | ______ | ______ |
| **4 _______** |  |  |  | ______ | | ______ | ______ | ______ | ______ | ______ |
| **5 _______** |  |  |  |  | | ______ | ______ | ______ | ______ | ______ |
| **6 _______** |  |  |  |  | |  | ______ | ______ | ______ | ______ |
| **7 _______** |  |  |  |  | |  |  | ______ | ______ | ______ |
| **8 _______** |  |  |  |  | |  |  |  | ______ | ______ |
| **9 _______** |  |  |  |  | |  |  |  |  | ______ |

Section. Discrimination

**These next questions are not directly about your health, but about other experiences you may have had in general. These are also important for understanding why women may have different health outcomes.**

**D1.** **First, I would like you to think about situations where you have been treated unfairly over your entire lifetime.**

*(Read and circle one response for each)*

| **Over your entire lifetime, how often….** | **Never** | **Rarely** | **Sometimes** | **Often** | **REF** | D/K |
| --- | --- | --- | --- | --- | --- | --- |
| 1. **have you been treated unfairly at school?**   ***READ “****Would you say Never, Rarely, Sometimes or Often”* ***after the first 2 questions and then as necessary****.* | 1 | 2 | 3 | 4 | 88 | 99 |
| 1. **have you been treated unfairly when getting hired or getting a job?** | 1 | 2 | 3 | 4 | 88 | 99 |
| 1. **have you been treated unfairly at work?** | 1 | 2 | 3 | 4 | 88 | 99 |
| 1. **have you been treated unfairly when getting housing?** | 1 | 2 | 3 | 4 | 88 | 99 |
| 1. **have you been treated unfairly when getting medical care?** | 1 | 2 | 3 | 4 | 88 | 99 |
| 1. **have you been treated unfairly when getting credit, bank loans, or a mortgage?** | 1 | 2 | 3 | 4 | 88 | 99 |
| 1. **have you been treated unfairly when seeking legal services related to immigration?** | 1 | 2 | 3 | 4 | 88 | 99 |
| 1. **have you been treated unfairly from the police or in the courts?** | 1 | 2 | 3 | 4 | 88 | 99 |

*If* ***ALL*** *“1,” “88,” or “99” Go to* ***D4***

*If* ***ANY*** *“2,” “3,” or “4” Go to* ***D2***

**D2.** **Please see Show Card #11. I will read through each option. Please tell me which of these may be the reasons why you were treated unfairly over your lifetime?**

(*Read and circle one response for each)*

|  | **Was it because of…** | **Yes** | **No** | REF | D/K |
| --- | --- | --- | --- | --- | --- |
| **a.** | **Your health insurance (or lack of)** | 1 | 2 | 88 | 99 |
| **b.** | **The way you speak English** | 1 | 2 | 88 | 99 |
| **c.** | **Your birthplace** | 1 | 2 | 88 | 99 |
| **d.** | **Your gender** | 1 | 2 | 88 | 99 |
| **e.** | **Your race/ethnicity** | 1 | 2 | 88 | 99 |
| **f.** | **Your age** | 1 | 2 | 88 | 99 |
| **g.** | **Your religion** | 1 | 2 | 88 | 99 |
| **h.** | **Your height or weight** | 1 | 2 | 88 | 99 |
| **i.** | **Your skin color** | 1 | 2 | 88 | 99 |
| **j.** | **Your sexual orientation** | 1 | 2 | 88 | 99 |
| **k.** | **Your education** | 1 | 2 | 88 | 99 |
| **l.** | **How much money you have** | 1 | 2 | 88 | 99 |
| **m.** | **A physical disability** | 1 | 2 | 88 | 99 |
| **n.** | **Your appearance on a given day** | 1 | 2 | 88 | 99 |
| **o.** | **Other:**  **please specify __________________** | 1 | 2 | 88 | 99 |

**D3. How stressful has this/have these experience(s) of unfair treatment usually been for you?**

(*Read responses and circle one)*

**1.  Not at all stressful**

**2.  A little stressful**

**3.  Somewhat stressful**

**4.  Extremely stressful**

88. REFUSED

99. D/K

SECTION: IMMIGRATION

**The following questions are about you and your family’s background and ancestry.**

**I1**. **In what US state or country were you born?**

**|__|** D/K **|__|** REFUSED

**I2. In what US state or country was:**

STATE COUNTRY D/K REF

- 1. **Your biological father born?**  ___________ _______ ­­___ ___
  2. **Your father’s mother (your grandmother) born?** ___________ _______ ____ ___
  3. **Your father’s father (your grandfather) born?**  ___________ ______ ___ ___
  4. **Your biological mother born?**  ___________ ______ ___ ___
  5. **Your mother’s mother (your grandmother) born?** ___________ _______ ___ ___
  6. **Your mother’s father (your grandfather) born?** ___________ _______ ___ ___

*If I1 is US-born, skip to I7. If not US-born, proceed to I3.*

**I3.** **How old were you when you first came to this country to live?**

____ ____ years old

88 REFUSED

99 D/K

**I4a. Have you lived ONLY in the United States during the past 12 months?**

**(READ RESPONSE CHOICES);**

**__ Yes, lived ALL 12 months in the United States (go to I5)**

**___ No, lived in the United states and another country or countries during the past 12 months (go to I4b)**

**___** D/K

**___** REFUSED

**I4b. How many months did you live in the United States?**

**_____ # mos**

**____** D/k

**____** REFUSED

**I4c In what other country or countries did you live and for how many months?**

**Country (specify) ____________ # mos ___**

**Country (specify) ____________ # mos ___**

**Country (specify) ____________ # mos ___**

___ D/K

___ REFUSED

**Next, we would like to know about which languages you speak and prefer.**

**15. What languages do you currently speak at home?**

____________________________________________ (*If English only, go to question I7)*

*___* D/K

*___* REFUSED

**I6. The next few questions are about speaking English.**

| *(Read responses and circle one response for each item*) | **Not at all well** | **Poorly** | **OK** | **Well** | **Very well** | REF | D/K |
| --- | --- | --- | --- | --- | --- | --- | --- |
| 1. **How well do you speak English?**   ***READ “****Not at all well, Poorly, OK, Well, Very well”* ***after the first 2 questions and then as necessary****.* | 1 | 2 | 3 | 4 | 5 | 88 | 99 |
| 1. **How well do you understand spoken English?** | 1 | 2 | 3 | 4 | 5 | 88 | 99 |
| 1. **How well do you read English?** | 1 | 2 | 3 | 4 | 5 | 88 | 99 |
| 1. **How well do you write English?** | 1 | 2 | 3 | 4 | 5 | 88 | 99 |

**I7. What languages did you speak before age 18?**

___________________________________­­­­­­­ 🡪*If more than one language Go to* ***a.*** *below:*

**___** D/K

**___** REFUSED

1. **Which language did you speak the most?**

**______________________________________________________________**

**___** D/K

**___** REFUSED

*If US born, go to Section.Occupation*

*All Else:*

**I8.** **Next, I would like to ask about your current level of stress [or the stress you feel] about living in the US. Please remember that any information you provide will be kept confidential. I will read some statements. Please respond ‘Never, Sometimes, Often, or Very Often’ after each.**

*(Read responses and circle one response for each item*)

| **You feel that living in the US is stressful:** | **Never** | **Sometimes** | **Often** | **Very  often** | N/A | REF | D/K |
| --- | --- | --- | --- | --- | --- | --- | --- |
| 1. **because you lack the opportunity to visit your country of origin.**   ***READ “****Would you say Never, Sometimes, Often, or Very Often”* ***after the first 2 questions and then as necessary****.* | 1 | 2 | 3 | 4 | 77 | 88 | 99 |
| 1. **because you are living away from your family, relatives, and friends.** | 1 | 2 | 3 | 4 | 77 | 88 | 99 |
| 1. **because you are unable to do the things you used to enjoy when you were in your country of origin.** | 1 | 2 | 3 | 4 | 77 | 88 | 99 |
| 1. **because you are mistreated by others of your ethnic group.** | 1 | 2 | 3 | 4 | 77 | 88 | 99 |
| 1. **because you have or had a job that is below your experience and qualifications.** | 1 | 2 | 3 | 4 | 77 | 88 | 99 |
| 1. **because you are treated as an outsider by other Americans.** | 1 | 2 | 3 | 4 | 77 | 88 | 99 |
| 1. **because you have few, if any, opportunities to participate in American politics.** | 1 | 2 | 3 | 4 | 77 | 88 | 99 |
| 1. **because you are constantly reminded of your minority status.** | 1 | 2 | 3 | 4 | 77 | 88 | 99 |
| 1. **because you are disappointed that your standard of living is not what you had hoped for when you first came to the US.** | 1 | 2 | 3 | 4 | 77 | 88 | 99 |
| **j. because you have to depend on others for understanding how to access support services that you need.** | 1 | 2 | 3 | 4 | 77 | 88 | 99 |
| **k. because you don't understand the healthcare system here in the U.S.** | 1 | 2 | 3 | 4 | 77 | 88 | 99 |
| **l. because you feel you have heavy responsibilities for BOTH your family here in the U.S. as well as family in your country of origin.** | 1 | 2 | 3 | 4 | 77 | 88 | 99 |
| m. **because you worry about losing ties to your parents’ culture.** | 1 | 2 | 3 | 4 | 77 | 88 | 99 |
| n. **because you feel you are obligated to take care of your parents in their old age.** | 1 | 2 | 3 | 4 | 77 | 88 | 99 |

I9. **These next few questions are about some immigration experiences you may have had.**

*(Read questions and circle one response for each item*)

|  | **Yes** | **No** | N/A | REF | D/K |
| --- | --- | --- | --- | --- | --- |
| a. **Do you feel guilty for leaving family or friends in your country of origin?** | 1 | 0 | 77 | 88 | 99 |
| b. **Have you been questioned about your legal status?** | 1 | 0 | 77 | 88 | 99 |
| c. **Do you think you will be deported if you go to a social or government agency, regardless of your legal status?** | 1 | 0 | 77 | 88 | 99 |
| d. **Do you avoid seeking health services due to fear of immigration officials, regardless of your legal status?** | 1 | 0 | 77 | 88 | 99 |

Section. Occupation

**The next set of questions are about your job or occupation.**

If I3 (age came to the US) age>21 then go to O1, else go to O8

**O1. First, we would like to know about your most recent job or occupation before you came to the US to live. In what kind of business or industry did you work? _______________________________**

(For example: health care, banking, education, manufacturing, retail)

__ did not work before coming to the US--> *go to O8*

**___** D/K

**___** REFUSED

**O2. What kind of work did you do or what was your job title?** ______________________________

(For example: registered nurse, personnel manager, supervisor of order department, machine operator.)

**___** D/K

**___** REFUSED

**O3**. **On average, how many hours did you work each week at (that/those) job(s) or occupation(s)?** __________________________ (hours/week)

**___** D/K

**___** REFUSED

**O4. Please look at Show Card #6. Which of the choices on that card best describes the hours you usually worked at (that/those) job(s) or occupation(s)?**

*If the respondent says “Flextime”, etc., probe to determine whether the shift that is worked actually falls in a day, evening, night, or rotating shift category before coding it as “another schedule.”*

| **Code or Value** | **Value Description** |
| --- | --- |
| **1** | A regular daytime schedule |
| **2** | A regular evening shift |
| **3** | A regular night shift |
| **4** | A rotating shift |
| **5** | Another schedule |
| **88** | Refused |
| **99** | Don't know |
|  |  |

**O5. About how long did you work at that job or business (in months/years)? _____ months/years**

**___** D/K

**___** REFUSED

**O6. Please look at Show Card #7. From that list of choices, how would you classify the skills required for that job? Would you say it,**

1. **required no formal education, on-the-job training is usually provided,**
2. **required some school education and/or occupation-specific training,**
3. **required college education or apprenticeship/trade training, or**
4. **required university education at the bachelor’s level or higher**
5. **___** D/K
6. **___** REFUSED

**O7. In that job, would you say your qualifications and skills were being…?**

*[Read categories to respondent].*

1. **Adequately used**
2. **Underused**
3. Refused

99. Don't know

**O8.** **Please look at Show Card #8. Which of the following best describes your usual employment status over the past 12 months**?

(*Circle One*)

| 1. | Working full-time |
| --- | --- |
| 2.  3.  4.  5.  6.  7.  8.  9.  87. | Working part-time  Unemployed or looking for work  Retired  On disability permanently  On disability for a period of time (on sick leave or maternity leave or disability leave for other reasons)  Keeping house/homemaker  Student  Volunteer/work without pay  Other, specify: ­­­­­­­­­­­­______________________ |
| 88. | REFUSED |
| 99. | D/K |

**O9. The next questions are about your usual occupation [here in the United States]. This would be the longest held job that best describes the kind of work you do (did) [here]:**

*(Read below questions and enter responses)*

1. **In what kind of business or industry do (did) you work?**

   __________________________________________________________
   (**For example:** health care, banking, education, manufacturing, retail)

**___** D/K

**___** REFUSED

b. **What kind of work do (did) you do or what was your job title?**
__________________________________________________________
(**For example:** registered nurse, personnel manager, supervisor of order department, machine operator.)

**___** D/K

**___** REFUSED

**O10.** **Please look at Show Card #6. Which of the choices on that card best describes the hours you usually work or worked at (that/those) job(s) or occupation(s)?**

*If the respondent says “Flextime”, etc., probe to determine whether the shift that is worked actually falls in a day, evening, night, or rotating shift category before coding it as “another schedule.”*

| **Code or Value** | **Value Description** |
| --- | --- |
| **1** | A regular daytime schedule |
| **2** | A regular evening shift |
| **3** | A regular night shift |
| **4** | A rotating shift |
| **5** | Another schedule |
| **88** | Refused |
| **99** | Don't know |
|  |  |

**O11.** **About how long did you work at that job or business (in months/years)?** _____ months/years

**___** D/K

**___** REFUSED

**O12.** **Please look at Show Card #7. From that list of choices, how would you classify the skills required for that job? Would you say, it ...**[showcard]

1. required no formal education, on-the-job training is usually provided
2. required some school education and/or occupation-specific training
3. required college education or apprenticeship/trade training
4. required university education at the bachelor’s level or higher
5. **___** D/K
6. **___** REFUSED

**O13. In that job, would you say your qualifications and skills were being…?**

*INTERVIEWER: Read categories to respondent.*

1. Adequately used
2. Underused

88. Refused

99. Don't know

Section. Early Life Exposures

**E1.** **How many brothers and sisters do you have that are older than you?** ___

**E2.** **How many brother and sisters do you have that are younger than you?** ___

**E3.** **Did you attend a preschool (that is, school before kindergarten, generally before age 5)?**

❑1 Yes

❑2 No

❑99 DK

❑88 REF

**E4**. Did you attend kindergarten or other school at age 5?

❑1 Yes

❑2 No

❑99 DK

❑88 REF

**E5.** **Before age 18, did you ever live in a dormitory?**

❑1 Yes 🡪 A. For how many years? 🞏🞏

❑2 No

❑99 DK

❑88 REF

**Next, I’m going to ask you some questions about your home and living environment at different ages of your life.**

**Let me ask about age…** [READ AGE AND PROCEED WITH QUESTIONS WORKING DOWN COLUMNS]

|  | Age | Age | Age | Past 12 months |
| --- | --- | --- | --- | --- |
| **At age XX ...or**  **[During the past 12 months ...]** | 6 months | 5 yrs | 12 yrs |  |
| **A. Please look at Show Card #9. Which of the choices on that card best describes the** | ❑1 Farm | ❑1 Farm | ❑1 Farm | ❑1 Farm |
| **area in which you lived: farm, rural, small** | ❑2 Rural | ❑2 Rural | ❑2 Rural | ❑2 Rural |
| **town, suburban, or urban/city?**  [showcard] | ❑3 Town | ❑3 Town | ❑3 Town | ❑3 Town |
|  | ❑4 Suburb | ❑4 Suburb | ❑4 Suburb | ❑4 Suburb |
|  | ❑5 Urban | ❑5 Urban | ❑5 Urban | ❑5 Urban |
|  | ❑99 DK  ❑88 REF | ❑99 DK  ❑88 REF | ❑99 DK  ❑88 REF | ❑99 DK  ❑88 REF |
| **B.** **Did your home have indoor plumbing, that is, running water?** | ❑1 Yes | ❑1 Yes | ❑1 Yes | ❑1 Yes |
|  | ❑2 No | ❑2 No | ❑2 No | ❑2 No |
|  | ❑99 DK  ❑88 REF | ❑99 DK  ❑88 REF | ❑99 DK  ❑88 REF | ❑99 DK  ❑88 REF |
| **C. On a scale of 1-5, 1 being lowest and 5** | ____ | ____ | ____ | ____ |
| **highest, rank your household’s general** |  |  |  |  |
| **financial situation** | ❑99 DK  ❑88 REF | ❑99 DK  ❑88 REF | ❑99 DK  ❑88 REF | ❑99 DK  ❑88 REF |
| **D. how** | ____ | ____ | ____ | ____ |
| **many siblings or other people usually** |  |  |  |  |
| **slept in the same bedroom as you?** | ❑99 DK  ❑88 REF | ❑99 DK  ❑88 REF | ❑99 DK  ❑88 REF | ❑99 DK  ❑88 REF |
| **E. Did you live within a half-mile of stables or** | ❑1 Yes | ❑1 Yes | ❑1 Yes | ❑1 Yes |
| **pens where horses, cows, pigs or other** | ❑2 No | ❑2 No | ❑2 No | ❑2 No |
| **hoofed animals were kept?** | ❑99 DK  ❑88 REF | ❑99 DK  ❑88 REF | ❑99 DK  ❑88 REF | ❑99 DK  ❑88 REF |
| **F.** **Did you help raise horses, cows, pigs, chickens, or other farm animals such that you had close contact with them and their feeding/sleeping areas?** | ❑1 Yes | ❑1 Yes | ❑1 Yes | ❑1 Yes |
|  | ❑2 No | ❑2 No | ❑2 No | ❑2 No |
|  | ❑99 DK  ❑88 REF | ❑99 DK  ❑88 REF | ❑99 DK  ❑88 REF | ❑99 DK  ❑88 REF |
| **H.** **Were you otherwise regularly exposed to animal feces (pet dogs, cats, rabbits, guinea pigs, etc)?** | ❑1 Yes  ❑2 No  ❑99 DK  ❑88 REF | ❑1 Yes  ❑2 No  ❑99 DK  ❑88 REF | ❑1 Yes  ❑2 No  ❑99 DK  ❑88 REF | ❑1 Yes  ❑2 No  ❑99 DK  ❑88 REF |

SECTION: DISCRIMINATION, DAY-TO-DAY

**Now I am going to ask you to think about situations where you have been treated unfairly again, but this time, please think about your everyday life. These are situations that may have happened over the past 12 months**.

**D4.** **How often have any of the following things happened to you in your day-to-day life?**

*(Read and circle one response for each)*

| **Over the past 12 months, how often….** | **Never** | **Rarely** | **Sometimes** | **Often** | **REF** | D/K |
| --- | --- | --- | --- | --- | --- | --- |
| 1. **have you been treated with less respect than other people?**   ***READ “****Would you say Never, Rarely, Sometimes or Often”* ***after the first 2 questions and then as necessary****.* | 1 | 2 | 3 | 4 | 88 | 99 |
| 1. **have you received poorer service than other people at restaurants or stores?** | 1 | 2 | 3 | 4 | 88 | 99 |
| 1. **have people acted as if they think you are not smart?** | 1 | 2 | 3 | 4 | 88 | 99 |
| 1. **have people acted as if they are afraid of you?** | 1 | 2 | 3 | 4 | 88 | 99 |
| 1. **have people acted as if they think you are dishonest?** | 1 | 2 | 3 | 4 | 88 | 99 |
| 1. **have people acted as if they’re better than you?** | 1 | 2 | 3 | 4 | 88 | 99 |
| 1. **have you been called names or were insulted?** | 1 | 2 | 3 | 4 | 88 | 99 |
| 1. **have you been threatened or harassed?** | 1 | 2 | 3 | 4 | 88 | 99 |
| 1. **have you been followed around in stores?** | 1 | 2 | 3 | 4 | 88 | 99 |

*Box* ***C***

*Box* ***B***

*Box* ***A***

*If* ***ALL*** *“88,” or “99” check box* ***C***

*If* ***ANY*** *“3,” or “4,” check box* ***B***

*If* ***2 or more*** *“2” check box* ***A***

*Check Boxes* ***A, B,*** *and* ***C****, page 24.*

*If Box* ***A OR*** *Box* ***B*** *checked, Go to* ***D5.***

*If Box* ***C*** *checked, determine why Refused* *or* D/K ***for all,*** *then Go to* ***Section.Reproductive History.***

*If* ***No Boxes*** *checked, Go to* ***Section.Reproductive History.***

**D5.** **Please see Show Card #11. I will read through each option. Please tell me which of these may be the reasons why you were treated unfairly?**

(*Read and circle one response for each)*

|  | **Was it because of…** | **Yes** | **No** | Refused | D/K |
| --- | --- | --- | --- | --- | --- |
| **a.** | **Your health insurance (or lack of)** | 1 | 2 | 88 | 99 |
| **b.** | **The way you speak English** | 1 | 2 | 88 | 99 |
| **c.** | **Your birthplace** | 1 | 2 | 88 | 99 |
| **d.** | **Your gender** | 1 | 2 | 88 | 99 |
| **e.** | **Your race/ethnicity** | 1 | 2 | 88 | 99 |
| **f.** | **Your age** | 1 | 2 | 88 | 99 |
| **g.** | **Your religion** | 1 | 2 | 88 | 99 |
| **h.** | **Your height or weight** | 1 | 2 | 88 | 99 |
| **i.** | **Your skin color** | 1 | 2 | 88 | 99 |
| **j.** | **Your sexual orientation** | 1 | 2 | 88 | 99 |
| **k.** | **Your education** | 1 | 2 | 88 | 99 |
| **l.** | **How much money you have** | 1 | 2 | 88 | 99 |
| **m.** | **A physical disability** | 1 | 2 | 88 | 99 |
| **n.** | **Your appearance on a given day** | 1 | 2 | 88 | 99 |
| **o.** | **Other:**  **please specify __________________** | 1 | 2 | 88 | 99 |

**D6.** **How stressful has this/have these experience(s) of unfair treatment usually been for you?**

*(Read responses and circle one)*

**1.  Not at all stressful**

**2.  A little stressful**

**3.  Somewhat stressful**

**4.  Extremely stressful**

88. REFUSED

99. D/K

D7.      **How did you respond to this/these experience(s)? Did you:**

            (*Read and circle one response for each)*

|  |  | **Yes** | **No** | **N/A** | **REF** | **D/K** |
| --- | --- | --- | --- | --- | --- | --- |
| a. | **try to do something about it** | 1 | 2 | 77 | 88 | 99 |
| b. | **accept it as a fact of life** | 1 | 2 | 77 | 88 | 99 |
| c. | **work harder to prove them wrong** | 1 | 2 | 77 | 88 | 99 |
| d. | **believe that you brought it on yourself** | 1 | 2 | 77 | 88 | 99 |
| e. | **talk to someone about how you were feeling** | 1 | 2 | 77 | 88 | 99 |
| f. | **express anger or get mad** | 1 | 2 | 77 | 88 | 99 |
| g. | **pray about the situation** | 1 | 2 | 77 | 88 | 99 |

Section. Reproductive History

**RH1.** **How many pregnancies have you had that have lasted at least 7 months?** |__|__|

**___** D/K

___ REFUSED

IF NONE SKIP TO RH4

**RH2.** **How old were you when your first child was born?** |__|__| AGE

**___** D/K

___ REFUSED

**RH3.** **Have you breast fed any of your children?**

|__| NO (GO TO RH4)

|__| YES --> **Please add together the number of months you breast fed each of your children. In total, for how many months have you breast fed?** |__|__|__|

**___** D/K

___ REFUSED

**RH4.** **How old were you when you had your first menstrual period?** ______ (age (years))

__ never had a period

888. Don’t know

999. Refused

**RH5.** **Did you ever have one or both of your ovaries removed? This may have been as part of a hysterectomy.**

0 NO (GO TO Section.Exogenous Hormones)

1 YES, ONE

2 YES, BOTH

9 DON'T KNOW (GO TO Section.Exogenous Hormones)

**___** REFUSED (GO TO Section.Exogenous Hormones)

**RH6.** **How old were you when you had your (ovary/ovaries) removed?**

|__|__| AGE 1ST OVARY REMOVED

|__|__| AGE 2ND OVARY REMOVED

**___** D/K

**___** REFUSED

Section. Exogenous Hormones

**ORAL CONTRACEPTIVES**

**H1.** **Have you used birth control pills (oral contraceptives) for one month or longer for any reason?** *(Circle one)*

1. No 🡪 Go to question H2
2. Yes
3. **___** D/K
4. **___** REFUSED

H1a. **How old were you when you first took birth control pills?** |__|__| AGE

**___** D/K

**___** REFUSED

H1b. **How old were you when you last took birth control pills?** |__|__| AGE

**___** D/K

**___** REFUSED

H1c. **Between ages XX and XX, have there been any months during which you did not use birth control pills?**

1. NO --> Go to question H2
2. YES
3. **___** D/K
4. **___** REFUSED

H1d. **During this time, for how any months or years did you stop using birth control pills?**

|__|__|__| MOS OR |__|__| YRS

**___** D/K

**___** REFUSED

**MENOPAUSE**

**H2.** **When was your last menstrual period? Please tell us the date or how old you were at the time:**

1. Month/Year: / Age: ___

**___** D/K

**___** REFUSED

**MENOPAUSAL HORMONE THERAPY**

**H3.** **Please look at Show Card #10. Which of the choices on that card best describe your current menstrual status:** *(Circle one)*

1. I am still having regular menstrual periods

2. I am not having regular menstrual periods because I am pregnant or breast-feeding (SKIP THE REMAINDER OF SECTION.EXOGENOUS HORMONES)

3. I am peri-menopausal, that is I am still having menstrual periods but they are not regular (they are heavy, continuous, or not occurring monthly)

4. I began taking hormone therapy while still having periods (GO TO H5)

5. My periods have stopped on their own, I had a natural menopause and did not take hormone therapy before my periods stopped

6. My periods have stopped after surgery to remove my uterus or both ovaries

7. My periods have stopped after radiation or chemotherapy

8. My periods have stopped for another reason. Please specify ____________________

**___** D/K

**___** REFUSED

**H4.** **Have you ever used menopausal hormone therapy?** *(Circle one)*

No 🡪 Go to the next section

Yes 🡪 Go to H5

**___** D/K

**___** REFUSED

**H5.** [ASK THE FOLLOWING FOR WOMEN USING menopausal hormone therapy:]

| **Which of the following types of pills have you used?** | | **IF USED: How many years in total did you use this type of pill (exclude any times when you stopped temporarily)** |
| --- | --- | --- |
| **estrogen-only pills, e.g., Premarin?** | 0 NO  1 YES  8 DON'T KNOW  9 REFUSED | \|__\|__\| YRS |
| **progesterone or progestin-only pills, such as Provera, Cycrin, medroxyprogesterone acetate, or Prometrium?** | 0 NO  1 YES  8 DON'T KNOW  9 REFUSED | \|__\|__\| YRS |
| **combination estrogen & progestin pills, e.g., Prempro, Premphase, or Femhrt?** | 0 NO  1 YES  8 DON'T KNOW  9 REFUSED | \|__\|__\| YRS |
| **other hormone pills?** (specify___________________) | 0 NO  1 YES  8 DON'T KNOW  9 REFUSED | \|__\|__\| YRS |

Section. Screening and Family History

**FH1.** **Have you ever had a screening mammogram?**

1. YES

2. NO

**___** D/K

**___** REFUSED

**FH2**. **When was your last screening mammogram?** ______________ (year)

**FH3.** **Over the past 10 years, on average, how often did you have a screening mammogram?**

1. **have had only 1 mammogram**

2. **every year**

3. **every 2 years**

4. **every 3 years**

5. **other** (specify)

6. D/K

7. REFUSED

**FH4.** **Did you ever have any of the following breast procedures: a fine needle aspiration (FNA), core biopsy, or surgical biopsy?**

1. YES (go to FH5)

2. NO (go to FH6)

3. D/K

4. REFUSED

**FH5.** If YES, **were you told you had a diagnosis of Atypical Hyperplasia or Atypia?**

1. YES

2. NO

3. D/K

4. REFUSED

| **FH6. Have any of your following relatives had breast cancer? Please include only biologic relatives, i.e., those related to you by blood.** | | IF YES: **Was your [RELATIVE} diagnosed with breast cancer before or after the age of 50?** |
| --- | --- | --- |
| **mother?** | 0 NO  1 YES  99 DON'T KNOW  88 REFUSED | 1 BEFORE AGE 50  2 AFTER AGE 50  99 DON'T KNOW  88 REFUSED |
| **sisters?** | 0 NO  1 YES  7 NO SISTERS  99 DON'T KNOW  88 REFUSED | SISTER #1:  1 BEFORE AGE 50  2 AFTER AGE 50  99 DON'T KNOW  88 REFUSED  SISTER #2:  1 BEFORE AGE 50  2 AFTER AGE 50  99 DON'T KNOW  88 REFUSED |
| **daughters?** | 0 NO  1 YES  7 NO DAUGHTERS  99 DON'T KNOW  88 REFUSED | 1 BEFORE AGE 50  2 AFTER AGE 50  99 DON'T KNOW  88 REFUSED |

Section. SocioDemographics

**SD1.** **Please look at Show Card #12. Which of the following best describes your marital or relationship status over the past 12 months?**

*(circle one)*

1. Legally married or have a registered domestic partner
2. Separated
3. Divorced
4. Widowed
5. Living with a partner to whom you are not married
6. In a relationship but not living with partner
7. Single
8. Other (please specify) ______________________
9. REFUSED

99. D/K

**SD2.** **Please look at Card Show Card #13. Over the past 12 months, what kind of place did you usually go to, or go to most often, when you were sick or needed advice about your health?**

1. doctor's office/kaiser/other hmo

2. hospital clinic

3. community or neighborhood clinic

4. emergency room

5. some other place (specify:_________)

6. no one place

88. refused

99. don't know

**SD3.** **Please look at Card #14. Over the past 12 months, did you have any of the following types of health insurance or health care coverage?**

*(Circle all that apply)*

1. Health insurance through my job or my husband’s/wife’s/partner’s job

(such as Blue Cross, HealthNet, Kaiser, etc.)

1. Individual health insurance not provided by my job or my husband’s/wife’s/partner’s job (such as Blue Cross, HealthNet, Kaiser, etc.)
2. MediCare Part A and/or Part B
3. MediCare Part D prescription drug coverage (MediCare drug card)
4. Extra insurance for MediCare (Medi-Gap)
5. Medi-Cal
6. Other government health program (county or state)
7. Military health care (such as TRICARE, VA, CHAMP-VA)
8. Indian Health Service
9. Single-service plan (such as dental, vision, prescriptions)

87. Other: _____________________________________

11. None of the above

88. REFUSED

99. D/K

**SD4*.*** **Please look at Show Card #15. What is the HIGHEST level of school you have completed or the highest degree you have received?**

*(Circle one)*

1. Never attended/kindergarten only
2. 1st grade
3. 2nd grade
4. 3rd grade
5. 4th grade
6. 5th grade
7. 6th grade
8. 7th grade
9. 8th grade
10. 9th grade
11. 10th grade
12. 11th grade
13. 12th grade, High School Graduate
14. 12^th^ grade, did not graduate
15. GED or took a test to graduate
16. Some college, no degree
17. Associate degree (such as AA, AS, ABA)
18. Bachelor's degree (such as BA, BS, BBA)
19. Master's degree (such as MA, MS, MBA)
20. Professional degree (such as MD, DDS, JD)
21. Doctoral degree (such as PhD, EdD)

88. REFUSED

99. D/K

**SD5.** **Did you ever go to school outside the United States (do not include study abroad programs)?** *[check one]*

- - Yes
- No → go to SD7
- D/K→ go to SD7
- REF→ go to SD7

**SD6**. **How many years of your education took place outside the United States?**

| **Elementary/Primary school** | __________  Years |
| --- | --- |
| **High school/Secondary school** | __________  Years |
| **University/Post secondary** | __________  Years |

**___** D/K

**___** REFUSED

**SD7.** **How many people are currently living in your household, including yourself?**

(*Write in number and follow skip pattern instructions*)

| 1. | Number of people _______ *If “1” Go to* ***SD8****, otherwise go to* ***2-4*** *below* |
| --- | --- |
| 2.  3.  4. | **Of these people, how many are children?** ­­­­________  **Of these people, how many are adults?** ________  **Of the adults, how many bring income into the household?** _________ |
| 88. | REFUSED *GO TO* ***SD8*** |
| 99. | D/K |

**SD8.** **Which of the following best describes the home where you live. Is it…?**

(*Read responses and circle one*)

1. Owned or being bought by you (or someone in the household)?

2. Rented for money?

3. Occupied without payment of money or rent?

4. Other (specify)_____________________________________

88. REFUSED

99. D/K

**SD9.** **Please look at Show Card #16. Which of these categories best describes your total combined family income for the past 12 months? This should include income (before taxes) from all sources, wages, rent from properties, social security, disability and/or veteran's benefits, unemployment benefits, workman's compensation, help from relatives (including child payments and alimony), and so on.**

(C*ircle one*)

| 1. | $24,000 or less |
| --- | --- |
| 2.  3.  4.  5.  6.  7.  8.  9.  10. | $25,000 through $35,000  $36,000 through $45,000  $46,000 through $55,000  $56,000 through $65,000  $66,000 through $75,000  $76,000 through $99,000  $100,000 through $149,000  $150,000 through $199,000  $200,000 or more |
| 88. | REFUSED |
| 99. | D/K |

Section: Survey Preference

**SP1. How often do you use the internet from a computer or a mobile device (like cell phone, ipad)?**

a. **never use**

b. **less than once a month**

c. **several times a month**

d. **several times a week**

e. **daily**

f. D/K

g. REF

**SP2.** **Would you be willing to complete a health survey such as this one on the internet?**

a. yes

b. no

c. D/K

d. REF

**SP3.** **What motivated you to participate in this health research?**  (*read response choices and circle all that apply*)

a. **in the hopes that my experience will help others**

b. **in the hopes that my experience will help my family members**

c. **for the monetary incentive**

d. **other reasons?** ________________________

e. D/K

f. REF

| **SP4. Would you be willing to provide any of the following for a health research study?** |  | **SP5.** **IF NO,** **what are your concerns about providing this type of sample?**  CHECK ALL THAT APPLY |
| --- | --- | --- |
| **a mouthwash sample or cheek swab using a q-tip** | \|__\| NO  \|__\| YES  \|__\| D/K  \|__\| REF | **\|__\| I worry my data will not be kept private**  **\|__\| I worry my data will be used to discriminate against me**  **\|__\| I am afraid of needles**  **\|__\| It goes against my culture**  **\|__\| other:** _________________________  \|__\| DON'T KNOW |
| **a blood sample (equivalent to about 3 tablespoons)** | \|__\| NO  \|__\| YES  \|__\| D/K  \|__\| REF | **\|__\| I worry my data will not be kept private**  **\|__\| I worry my data will be used to discriminate against me**  **\|__\| I am afraid of needles**  **\|__\| It goes against my culture**  **\|__\| other:** _________________________  \|__\| DON'T KNOW |
| **a tiny stool sample** | \|__\| NO  \|__\| YES  \|__\| D/K  \|__\| REF | **\|__\| I worry my data will not be kept private**  **\|__\| I worry my data will be used to discriminate against me**  **\|__\| I am afraid of needles**  **\|__\| It goes against my culture**  **\|__\| other:** _________________________  \|__\| DON'T KNOW |
| **a urine sample** | \|__\| NO  \|__\| YES  \|__\| D/K  \|__\| REF | **\|__\| I worry my data will not be kept private**  **\|__\| I worry my data will be used to discriminate against me**  **\|__\| I am afraid of needles**  **\|__\| It goes against my culture**  **\|__\| other:** _________________________  \|__\| DON'T KNOW |
|  |  | |
